# Discovery of Highly Potent BET Inhibitors based on a Tractable Tricyclic Scaffold

**DOI:** 10.1021/acsmedchemlett.4c00621

**Published:** 2025-03-21

**Authors:** Jaffer M. Zaidi, Eleonora Comeo, Andrew Baxter, Alex G. S. Preston, Weng C. Chan, Michael J. Stocks

**Affiliations:** † Biodiscovery Institute, School of Pharmacy, 6123 University of Nottingham, University Park, Nottingham NG7 2RD, U.K.; ‡ Medicines Research Centre, 1929 GSK, Gunnels Wood Road, Stevenage SG1 2NY, U.K.

**Keywords:** Bromodomain, BET inhibitor, BRD4

## Abstract

The bromodomain and extra-terminal domain (BET) protein family is a class of epigenetic reader proteins that recognize *N*-acetylated lysine residues in histone tails, playing a crucial role in gene expression and cell transcription. Selective inhibition of bromodomain-containing proteins (BRDs) disrupts transcription in key oncogenes. Over the past decade there has been considerable interest in developing small molecule BET inhibitors for the treatment of hematological malignancies and solid tumors. Herein, we report the development of a triazinoindole scaffold capable of the inhibition of bromodomain-containing protein 4 (BRD4), with either dimethylisoxazole or dimethyltriazole substituents acting as chemomimetics of the *N*-acetylated lysine residues. Derivatization of the parent scaffold afforded the lead compound, which displays low nanomolar affinity toward BRD4-BD1 with a favorable physicochemical and *in vitro* stability profile.

## Graphical abstract

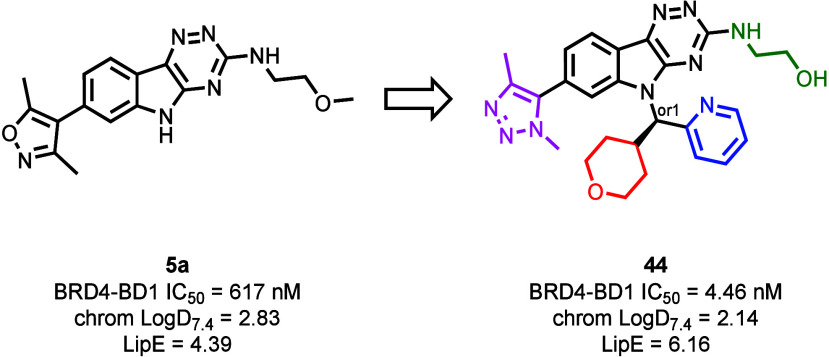

The bromodomain and extra-terminal domain (BET) protein family is composed of bromodomain-containing proteins 2, 3, and 4 (BRD2, BRD3 and BRD4 respectively) and bromodomain testis-specific protein (BRDT).^1^ These proteins are epigenetic readers that form multimolecular complexes with enzymes involved in the covalent modification of DNA and the proteins that package DNA, such as histones, and so play an important role in several physiological processes.^2^

The primary function of BETs is to regulate cell transcription by binding to *N*-acetylated lysine residues on histone tails.^3^ BET inhibitors prevent this key interaction in cancer cells, triggering the downregulation of certain genes and resulting in an overall reduction in gene expression.^4^ There has been extensive research in recent years, resulting in the successful development of over 20 small molecule BET inhibitors, with some of these now in clinical trials for the treatment of hematological malignancies and solid tumors.^5^ While preliminary results have confirmed the antitumor potential of BET inhibitors, several programs have also been terminated due to either safety concerns or limited monotherapeutic efficacy.^6^

There are several bioisosteres which can be incorporated into BET inhibitor scaffolds to displace *N*-acetylated lysine-mimicking peptides from bromodomains (BDs) (Figure 1).^7^ The first BET inhibitor was JQ1 (**1**), reported in 2010.^8^ This compound includes a methyltriazole moiety, which acts as an *N*-acetylated lysine mimetic. Other representative examples are the dimethylisoxazole-containing I-BET151 (**2**) and the pyridone-based ABBV-075 (**3**).^9,10^ Isoxazoles are of particular interest as *N*-acetylated lysine mimetics due to their structural simplicity and high lipophilic efficiency (LipE).^11^ However, it can be challenging to develop isoxazole-containing BET inhibitors with high potency, as seen in the case of **2**.^12^

**Figure 1:**
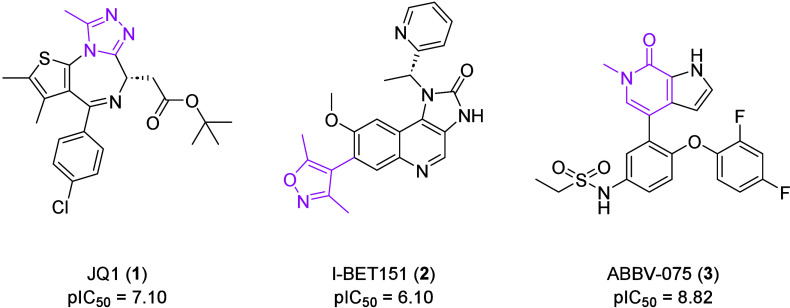
Structures and biological data of **1**, **2** and **3**. *N*-Acetylated lysine mimetics are highlighted in pink. Activity against BRD4 of **1** was determined by AlphaScreen, **2** by fluorescence polarization (FP) and **3** by fluorescence resonance energy transfer (FRET) binding.^8,10,13^

Compounds **1**, **2** and **3** effectively downregulate cellular myelocytomatosis (c-Myc) expression and inhibit tumor cell proliferation when used to treat advanced malignancies such as AML.^14–16^ Unfortunately, **1** had low oral bioavailability and overly short half-life of 1 h and was therefore unable to progress to clinical trials.^17^ **2** exhibited cardiotoxicity in Phase I studies and **3** is currently undergoing evaluation, underlining the difficulties in advancing BET inhibitors through clinical trials.^17–19^

The final BET inhibitor discussed is BMS-986158 (**4**) (Figure 2).^20^ **4** has high potency (pIC_50_ = 8.54) against BRD4-BD1, with a dimethyltriazole ring as the *N*-acetylated lysine mimetic. However, due to high lipophilicity (cLogP ∼ 5.0), it has a low LipE of 3.51.^21,22^ LipE is an important parameter used in drug discovery linking potency and lipophilicity and is calculated from pIC_50_ minus cLogP.^23^ Empirical evidence suggests that drug candidates have a LipE > 6, obtained by increasing potency while decreasing lipophilicity during lead optimization giving compounds with better pharmacological properties, such as improved solubility and metabolic stability.^24^

**Figure 2:**
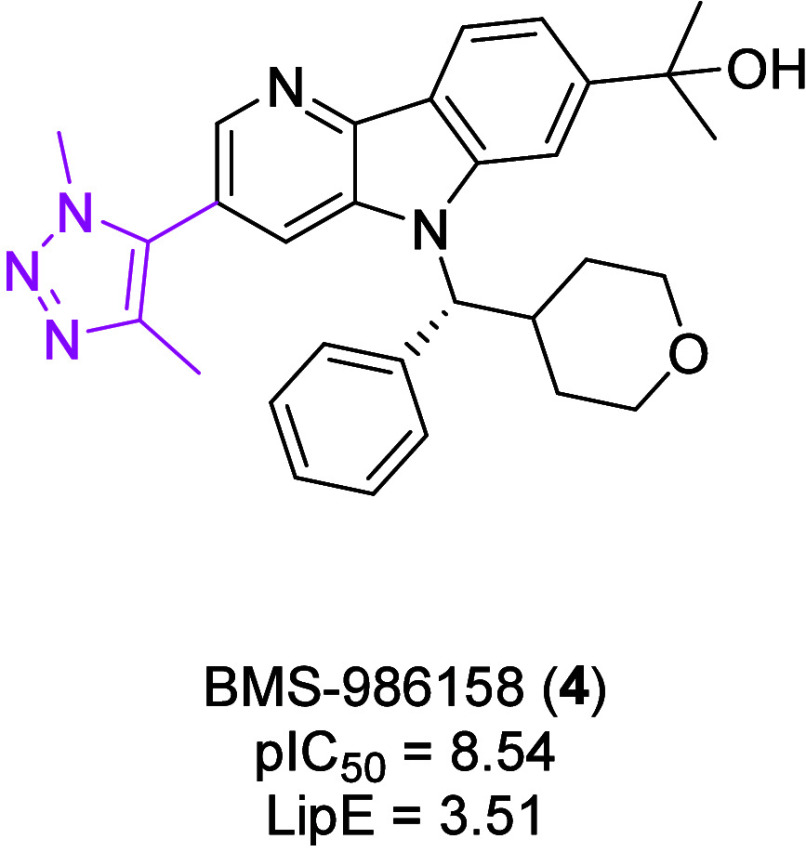
Structure and biological data of compound **4**. Potency measured against BRD4-BD1, as determined by FRET binding.^21^ LipE = pIC_50_ – cLogP, predicted using Data Warrior.^22,23^

Our aim was to develop a BET inhibitor with levels of potency comparable to those of **4** with reduced lipophilicity (increased LipE). A new tricyclic scaffold was prepared, and we report its optimization to highly potent and metabolically stable BET inhibitors.

We began with the identification of a triazinoindole core, inspiration of which originated from previous research carried out by Soukarieh which focused on developing novel treatments for *Pseudomonas aeruginosa*.^25^ The dimethylisoxazole moiety was incorporated as an *N*-acetylated lysine mimetic, located at either the 7- (**5a**) or 8- (**5b**) positions (Figure 3).^7^ The 2-methoxyethylamine group on the triazine was included to aid in aqueous solubility. Docking analysis of the BRD4-BD1 binding pocket confirmed that the WPF shelf, an important tryptophan-proline-phenylalanine sequence found in all BRDs, could be accessed from functionalization of the indole nitrogen given the structural similarity of **5** to benchmark **4** (Figures S1–2).^20,26^

**Figure 3:**
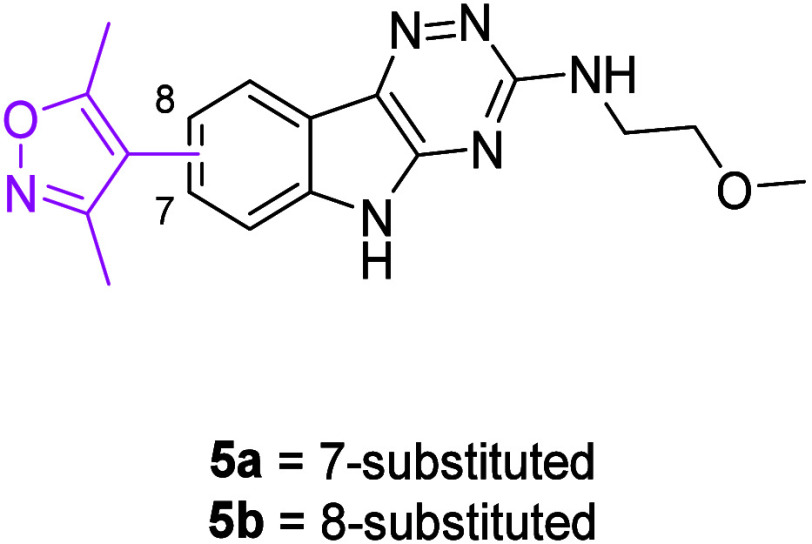
Structure of compounds **5a**-**b**.

The synthesis of **5a**-**b** commenced with acetal protection of either 6-bromoisatin (**6a**) or 5-bromoisatin (**6b**), followed by Suzuki–Miyaura cross-coupling with 3,5-dimethylisoxazole-4-boronic acid pinacol ester (Bpin) to afford biaryls (**8a**-**b**) (Scheme 1). Acid-mediated deprotection gave isatins (**9a**-**b**). Cyclization of these with thiosemicarbazide formed triazines (**10a**-**b**) and *S*-methylation yielded thioethers (**11a**-**b**). A one-pot oxidation and displacement process provided **5a**-**b**. *N*-Alkylations using the requisite alkyl halide gave *N*-alkylindoles (**12**-**13**) (Scheme 2).

**Scheme 1:**
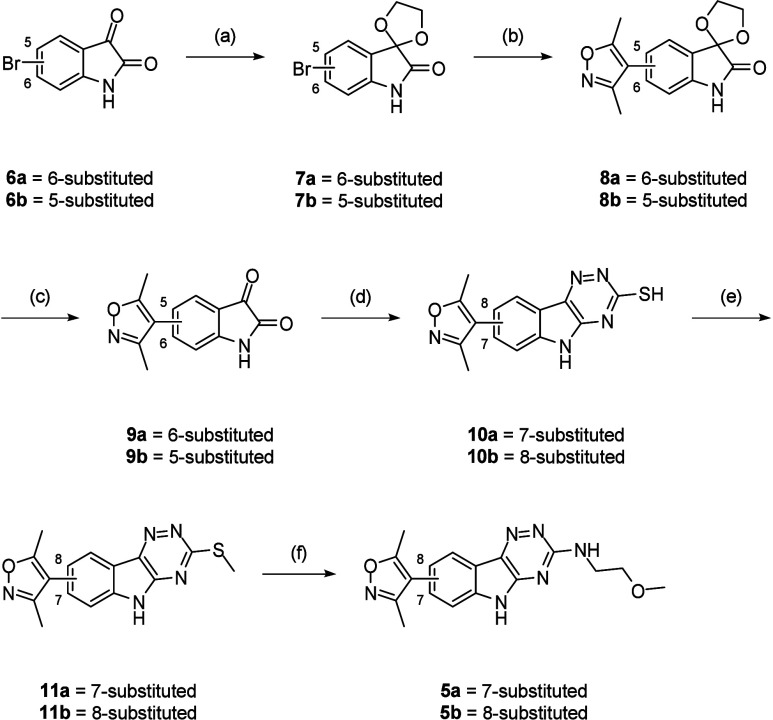
Synthesis of **5**–**11** ^a^

a Reagents and conditions: (a) HO­(CH_2_)_2_OH, *p*-TsOH·H_2_O, PhMe, reflux, 16–64 h, 50–54%; (b) 3,5-dimethylisoxazole-4-Bpin, Pd­(dppf)­Cl_2_·DCM, K_3_PO_4_ (aq), 2-MeTHF, 85 °C, 1 h, 51–54%; (c) AcOH, 12 M HCl, rt to 90 °C, 0.25–1 h, 64%; (d) thiosemicarbazide, K_2_CO_3_, H_2_O, 110 °C, 2–3 h, 55–64%; (e) MeI, Et_3_N, DMF, rt, 17–18 h, 71–80%; (f) (i) *m*-CPBA, NMP, rt, 1 h; (ii) OMe­(CH_2_)_2_NH_2_, DIPEA, 110 °C, 2 h, 44–73%.

**Scheme 2:**
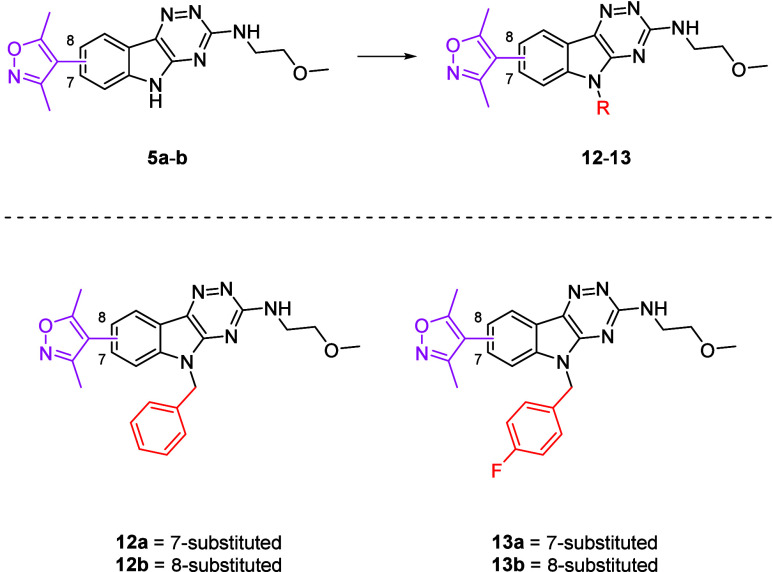
Synthesis of **12**-**13** ^a^

a Reagents and conditions: alkyl halide, K_2_CO_3_, DMF, rt to 70 °C, 17–19 h, 63–72%.

**5a**-**b** and **12**-**13** were submitted for biological evaluation with three main metrics measured, potency (pIC_50_), LipE and property forecast index (PFI) (Table 1). Potency was determined against BRD4-BD1 using FRET binding, with this being treated as a surrogate of BRD4 activity given the homology between BD1 and BD2 across the BET proteins.^21,27^ PFI is calculated from hydrophobicity plus aromatic ring count (chromatographic (chrom) logD_7.4_ + #Ar), with this metric being indicative of developability, or likelihood of progression to a drug candidate.^28^ PFI was used to assess the physicochemical profile of compounds, with a target of PFI < 7 as risks for several parameters, including solubility, are increased above this upper limit.^29^ Our objective was to develop a BET inhibitor with high potency and favorable physicochemical properties, as determined using PFI and LipE to guide compound optimization.

**Table 1:** Biological Data for **5** and **12**-**13**

Compound	pIC_50_ ± SD^a^	PFI^b^	LipE^c^
**5a**	6.22 ± 0.05	6.83	4.39
**5b**	5.64 ± 0.05	6.78	3.82
**12a**	7.53 ± 0.12	10.6	4.22
**12b**	6.08 ± 0.34	10.8	2.77
**13a**	7.34 ± 0.04	10.7	3.93
**13b**	6.18 ± 0.05	10.8	2.77

a Potency (*n* = 4) against BRD4-BD1, as determined by FRET binding.^21^ SD = standard deviation.

b PFI = chrom LogD_7;4_ + #Ar.^28^

c LipE = pIC_50_ – cLogP, predicted using Data Warrior.^22,23^

Initial results showed the 7-substituted analogues displayed higher biological activities than their 8-substituted counterparts, with this difference amplified upon further functionalization. **5a** was approximately four times more potent than its matched pair **5b** (pIC_50_ = 6.22 for **5a**, versus 5.64 for **5b**). Compounds **12**-**13** all demonstrated submicromolar activity against BRD4-BD1, and this observation was consistent with literature precedent that a lipophilic functional group in the vicinity of the WPF shelf is optimal for binding to the BRD.^30^ Unfortunately, the high lipophilicity of these molecules meant that they had PFI > 7 and so were likely to have solubility issues.^28^

We focused on reducing PFI and increasing LipE to more acceptable levels, and this was achieved by attaching a branched substituent containing an aromatic group to boost potency, through interaction with the WPF shelf, and an aliphatic group to lower lipophilicity, measured as chrom LogD_7.4_. The method of *N*-functionalization employed was a Mitsunobu reaction, and preparation of the corresponding alcohols was achieved in two steps. The unsymmetrical ketone precursors (**14**-**17**), obtained from a reaction between the constituent aliphatic ketones and aryl aldehydes, were reduced to the alcohols (**18**-**21**) (Scheme 3).^31^ Subsequent Mitsunobu reactions gave *N*-alkylindoles (**22**-**26**) (Scheme 4). Given the differences in experimental potency, priority was given to synthesizing 7-substituted analogues, derived from **5a**. In the cases of **25** and **26**, the Boc protecting groups were removed to give *N*-alkylindoles (**27**-**28**).

**Scheme 3:**
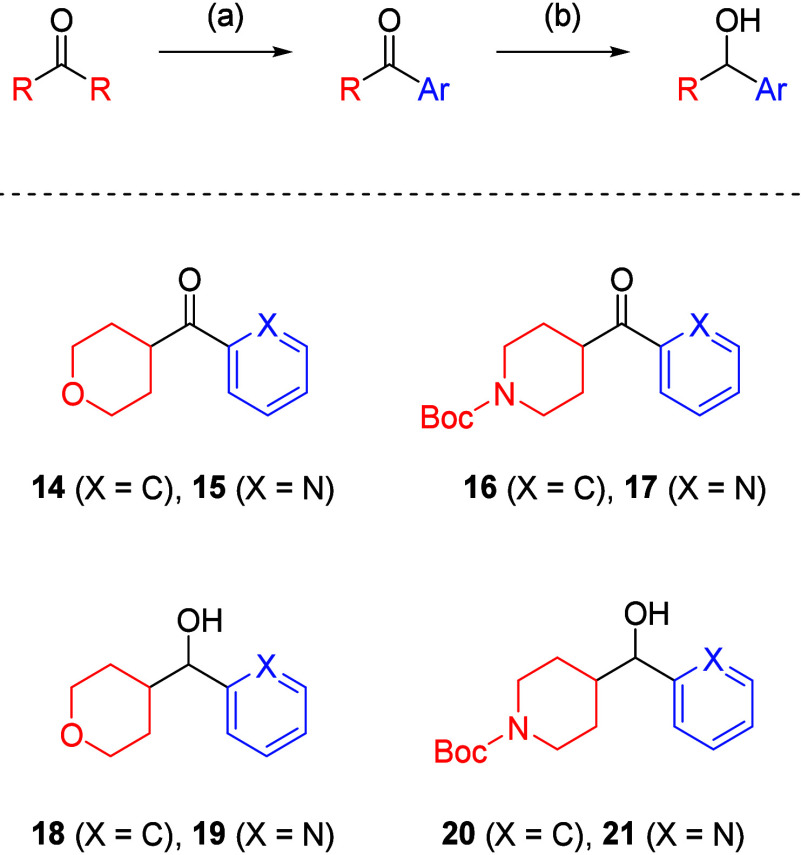
Synthesis of **14**-**21** ^a^

a Reagents and conditions: (a) (i) TsNHNH_2_, MeOH, rt, 2–2.5 h; (ii) aryl aldehyde, Cs_2_CO_3_, dioxane, reflux, 16–41 h, 47–66%; (b) NaBH_4_, MeOH, 0 °C to rt, 2 h, 66–99%.

**Scheme 4:**
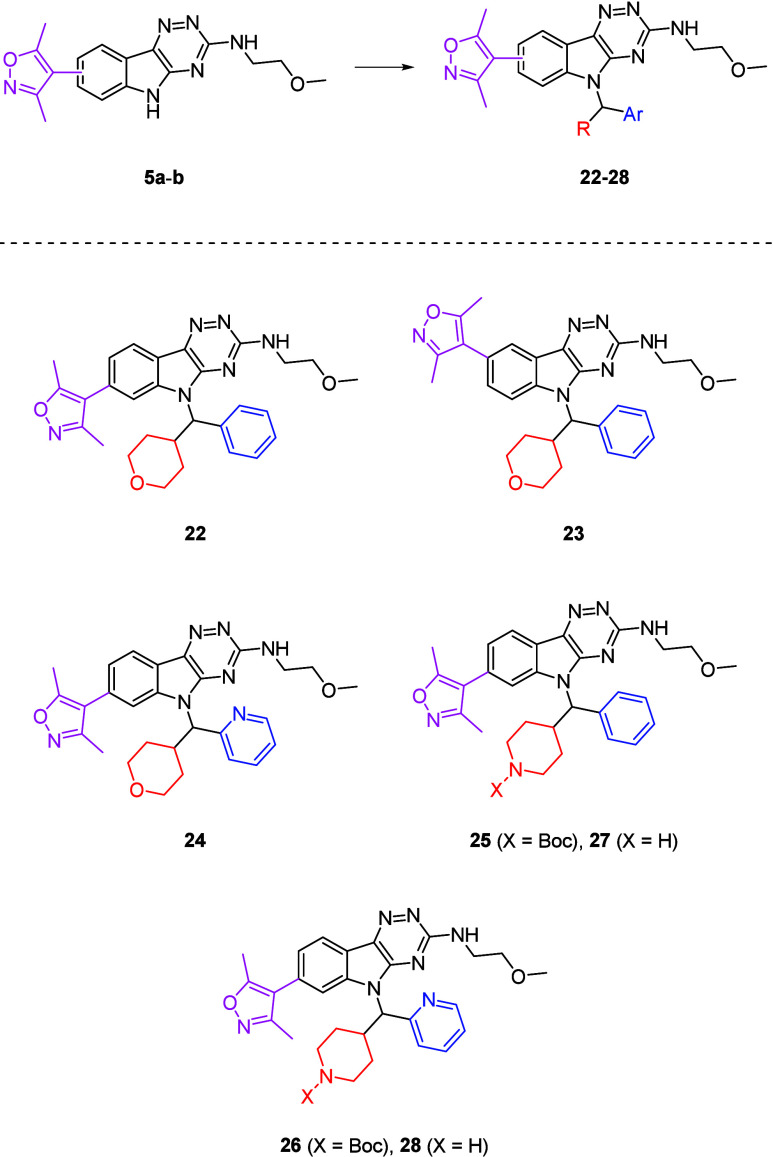
Synthesis of **22**–**28** ^a^

a Reagents and conditions: (i) alcohol, DIAD, PPh_3_, PhMe, rt, 3–22 h, 19–81%; (ii) 4.0 M HCl, dioxane, rt, 0.25–2 h, 35–81% (for **25** and **26** only).

Compounds **22**-**24** and **27**-**28** were biologically evaluated (Table 2). Some compounds proved potent (IC_50_ < 10 nM), an encouraging feat given the previously outlined difficulties in developing isoxazole-containing BET inhibitors with potencies above the micromolar range.^12^ **28** represented the most favorable balance of potency, PFI and LipE (pIC_50_ = 7.74, PFI = 7.37 and LipE = 4.25). The incorporation of a piperidine moiety proved beneficial as a chrom LogD_7.4_ modulator with the PFI closer to 7. Effort was now focused on reducing PFI < 7 and increasing LipE in **28**.

**Table 2:** Biological Data for **22**-**24** and **27**-**28**

Compound	pIC_50_ ± SD^a^	PFI^b^	LipE^c^
**22**	8.27 ± 0.31	10.6	3.69
**23**	6.33 ± 0.03	10.7	1.75
**24**	8.01 ± 0.11	9.74	4.38
**27**	7.63 ± 0.05	7.96	3.20
**28**	7.74 ± 0.12	7.37	4.25

a Potency (*n* = 4) against BRD4-BD1, as determined by FRET binding.^21^ SD = standard deviation.

b PFI = chrom LogD_7;4_ + #Ar).^28^

c LipE = pIC_50_ – cLogP, predicted using Data Warrior.^22,23^

Two structural modifications to **28** proposed to further lower lipophilicity were to replace the methoxy ethylamine with a hydroxy ethylamine and change the lipophilic dimethylisoxazole for a more polar dimethyltriazole. This led to the design of *N*-alkylindoles (**29**-**30**) (Figure 4). The isoxazole-triazole switch was not expected to impact potency given the similarity in binding modes between the two *N*-acetylated lysine mimetics with BRD4-BD1, with the same strategy adopted by Gavai during the development of **4**.^20^

**Figure 4:**
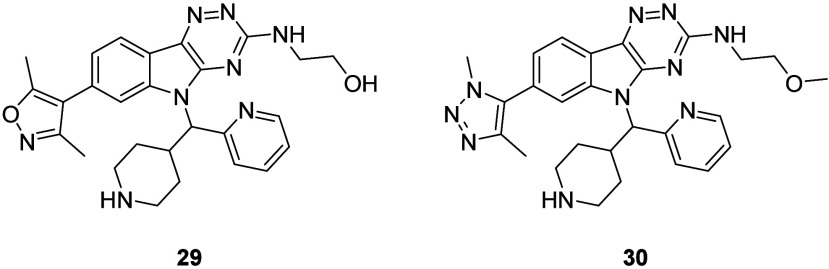
Structures of **29**-**30**.

Demethylation of **28** to produce **29** was not possible due to difficulties in carrying out these reactions on aliphatic methyl ethers.^32^ Instead, the synthetic route that was previously used in the preparation of **5a**-**b** was used here to make **11a**, at which point a Mitsunobu reaction with **21** was carried out to attain *N*-alkylindole (**31**) (Scheme 5). This was subjected to one-pot oxidation and displacement with ethanolamine, followed by Boc deprotection, and gave **29**.

**Scheme 5:**
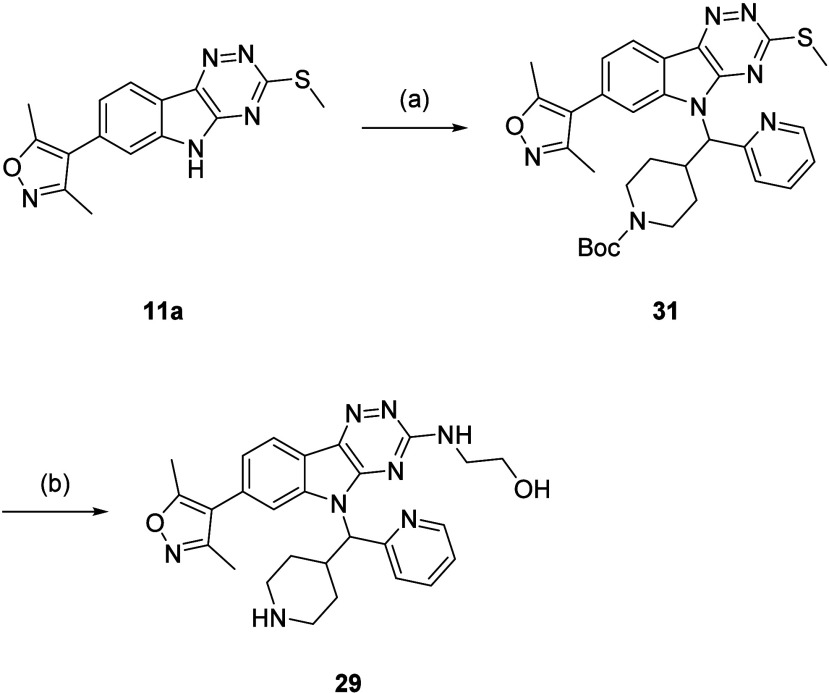
Synthesis of **29** and **31** ^a^

a Reagents and conditions: (a) **21**, DIAD, PPh_3_, PhMe, rt, 18 h, 71%; (b) (i) *m*-CPBA, NMP, rt, 1 h; (ii) HO­(CH_2_)_2_NH_2_, DIPEA, 180 °C, 2 h; (iii) 4.0 M HCl, dioxane, rt, 3 h, 4%.

For **30**, dimethyltriazole (**32**) was required. A one-pot, three-component coupling of 1,1-dimethoxyacetone (**33**), TsNHNH_2_ and MeNH_2_ yielded dimethyltriazole (**34**), which was converted to **32** through lithiation and reaction with Bu_3_SnCl (Scheme 6).^33^ Stille coupling of **7a** with **32** afforded **35** and acid-mediated acetal deprotection yielded isatin (**36**). Cyclization with thiosemicarbazide formed triazine (**37**), which was *S*-methylated to give thioether (**38**). A one-pot oxidation and displacement with 2-methoxyethylamine produced an amine (**39**). A Mitsunobu reaction of **39** with **21**, followed by the final Boc deprotection, gave **30**.

**Scheme 6:**
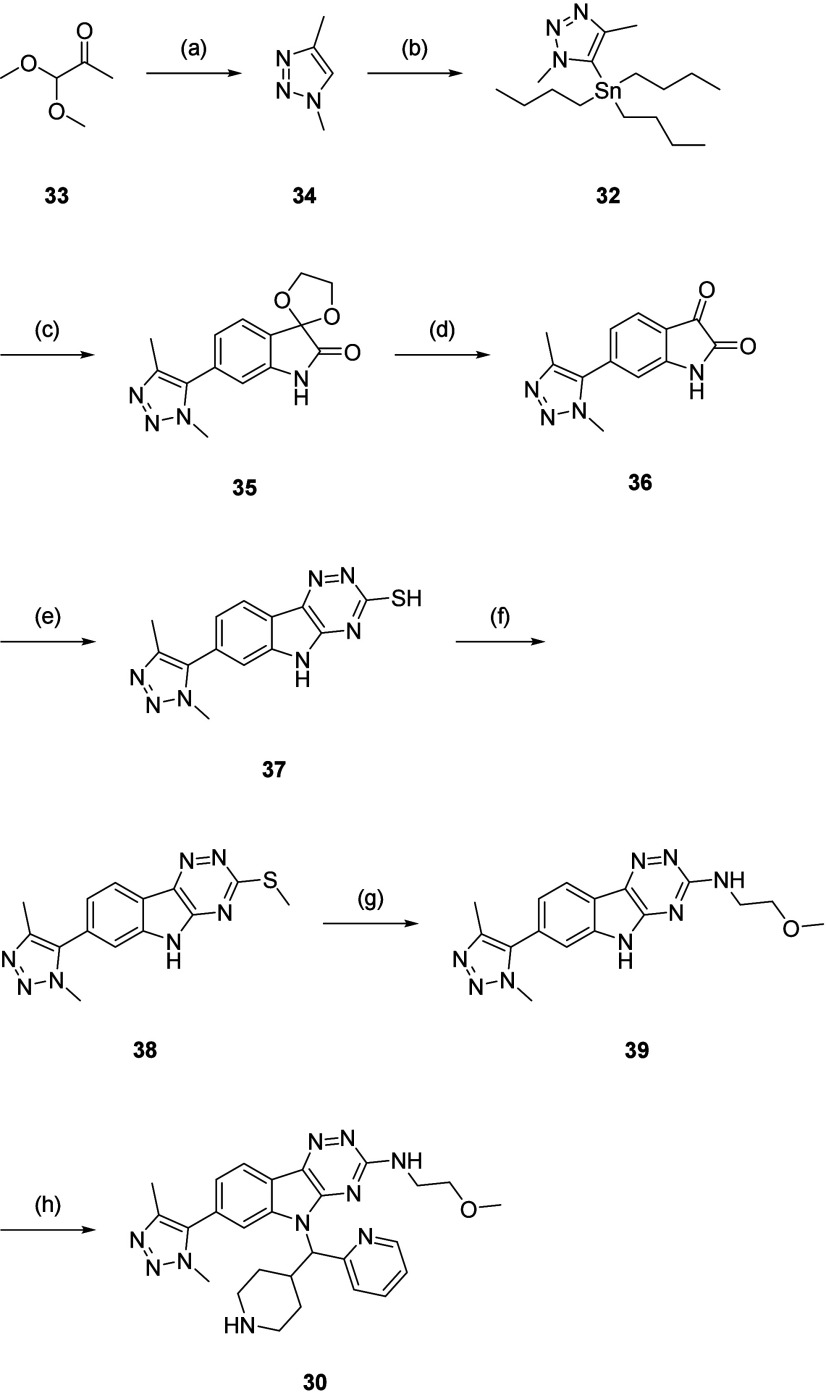
Synthesis of **30** and **32**-**39** ^a^

a Reagents and conditions: (a) (i) TsNHNH_2_, MeOH, rt, 0.1 h; (ii) MeNH_2_, Et_3_N, 140 °C, 0.1 h, 89%; (b) Bu_3_SnCl, *n*-BuLi, THF, −78 °C to rt, 1 h, 83%; (c) **7a**, Pd­(PPh_3_)_4_, CuI, Et_3_N, DMF, 95 °C, 2 h, 70%; (d) AcOH, 12.0 M HCl, rt, 4 h, 39%; (e) thiosemicarbazide, K_2_CO_3_, H_2_O, 110 °C, 2 h, 65%; (f) MeI, Et_3_N, DMF, rt, 16 h, 73%; (g) (i) *m*-CPBA, NMP, rt, 1 h; (ii) MeO­(CH_2_)_2_NH_2_, DIPEA, 110 °C, 2 h, 31%; (h) (i) **21**, DIAD, PPh_3_, PhMe, rt, 20 h; (ii) TFA, DCM, rt, 2 h, 6%.

The biological data for **29** and **30** was encouraging, with both demonstrating high potencies against BRD4-BD1 (pIC_50_ = 7.29 ± 0.06 for **29** (LipE = 4.23) and 7.41 ± 0.03 for **30** (LipE = 4.93)). The PFI was also <7 (PFI = 6.74 for **29** and 6.46 for **30**), indicating that the structural modifications to the core scaffold had been beneficial. Additionally, the *in vitro* permeability of these compounds was determined by a parallel artificial membrane permeability assay (PAMPA).^34^ Unfortunately, both molecules possessed very low permeabilities (<3.00 nm/s), which was hypothesized to be due to the basic piperidine moiety. This would be protonated at physiological pH and decrease the overall affinity of the compound toward the hydrophobic membrane region.^35^

The emphasis switched to improving permeability, and this was achieved by combining the moieties which were expected to give the best balance of potency and physicochemical properties. The hydroxy ethylamine side chain in **29** and triazole warhead in **30** were incorporated, and the piperidine moiety was replaced with a polar nonbasic THP ring to give *N*-alkylindole (**40**) (Figure 5). Given the permeability of **4** (498 nm/s), which has a similar core, and the potency of our closest matched pair **24** (pIC_50_ = 8.01), which contains the same WPF shelf group, we were sufficiently encouraged that these structural changes would increase permeability without compromising potency.

**Figure 5:**
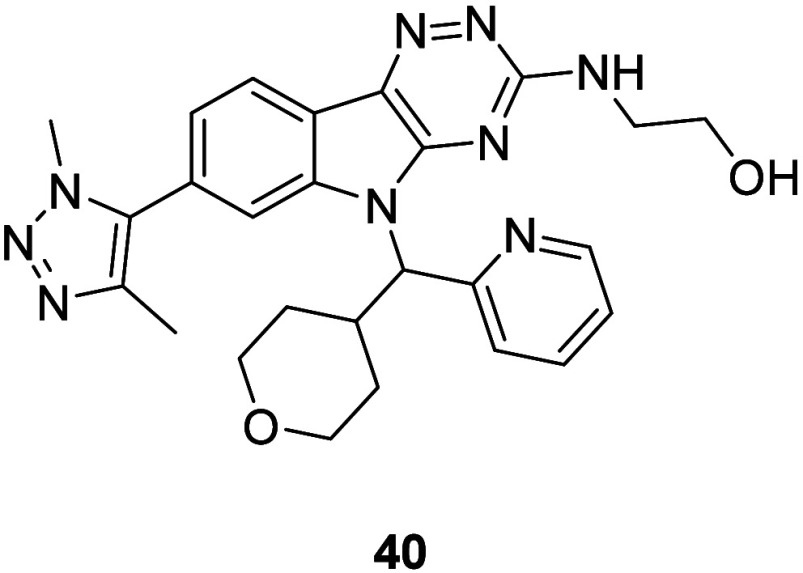
Structure of compound **40**.

The synthesis of **40** started from **6a**, with cyclization using thiosemicarbazide forming triazine (**41**) (Scheme 7). *S*-Methylation gave thioether (**42**), and a Mitsunobu reaction yielded *N*-alkylindole (**43**). One-pot oxidation and displacement with ethanolamine were followed by Stille coupling using **32** to afford **40**.

**Scheme 7:**
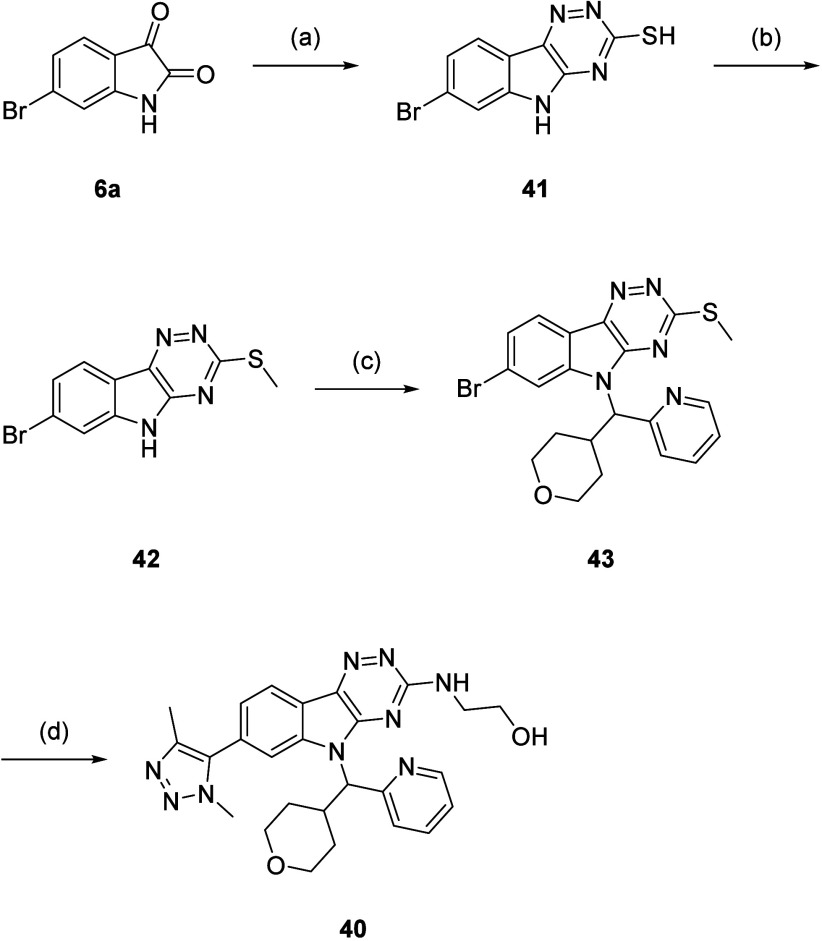
Synthesis of **40**–**43** ^a^

a Reagents and conditions: (a) thiosemicarbazide, K_2_CO_3_, H_2_O, reflux, 16 h, 90%; (b) MeI, Et_3_N, DMF, rt, 20 h, 73%; (c) **19**, DIAD, PPh_3_, PhMe, rt, 17 h, 46%; (d) (i) *m*-CPBA, NMP, rt, 1 h; (ii) HO­(CH_2_)_2_NH_2_, DIPEA, 180 °C, 1 h; (iii) **32**, Pd­(PPh_3_)_4_, CuI, Et_3_N, DMF, 95 °C, 1 h, 22%.

Compound **40** showed increased potency and LipE compared to **29** and **30** (pIC_50_ = 7.98 ± 0.17 (LipE = 5.79) for **40**, compared to 7.29 (LipE = 4.23) and 7.41 (LipE = 4.93) for **29** and **30**, respectively). As expected, there was a slight increase in PFI due to the piperidine-THP switch (PFI = 7.15 for **40**, compared to 6.74 and 6.46 for **29** and **30**, respectively). The permeability of **40** was significantly better (104 nm/s, compared to <3.00 nm/s for both **29** and **30**), due to the reduced hydrogen bond donor count and absence of ionizable center in **40** compared to **29** and **30**. These results provided validation that racemate **40** was the lead compound as it had the best balance of potency and physicochemical properties, and this was purified by chiral HPLC (CHIRALPAK IB N-5 column) to yield enantiomers (**44**) and (**45**) (Figure 6).

**Figure 6:**
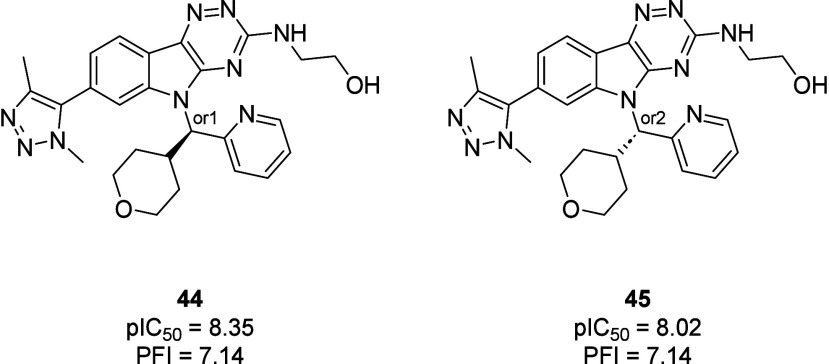
Structures and biological data for compounds **44** and **45**. Potency (*n* = 2) against BRD4-BD1, as determined by FRET binding.^21^ PFI = chrom LogD_7;4_ + #Ar.^28^

Compound **44** (pIC_50_ = 8.35 ± 0.01) was more active compared to **45** (pIC_50_ = 8.02 ± 0.08), while the PFIs of both enantiomers were identical (PFI = 7.14). For both enantiomers, kinetic solubility, permeability and metabolic stability data, the latter measured by *in vitro* microsomal intrinsic clearance (CL_int_) in rats and humans, was collected (Table 3).^34,36,37^ Docking of **44** and **45** into the BRD4-BD1 binding pocket was also carried out (Figures S3–4).

**Table 3:** Additional Biological Data for **4**, **44, 45**

Metric		Compound	
	**4**	**44**	**45**
Solubility^a^ (μg/mL)	109	134	138
Permeability^b^ (nm/s)	498	77.8	93.6
Rat CL_int_ ^c^(μL/min/mg)	ND^f^	<10.0	<10.0
Human CL_int_ ^c^ (μL/min/mg)	ND^f^	<10.0	<10.0
PFI^d^	9.08	7.14	7.14
LiPE^e^	3.51	6.16	5.83

a Solubility (*n* = 1) is determined by CAD.^36^

b Permeability (*n* = 2) is determined by PAMPA.^34^

c CL_int_ (*n* = 1) is determined by *in vitro* metabolic stability in liver microsomes.^37^

d PFI = chrom LogD_7;4_ + #Ar.^28^

e LipE = pIC_50_ – cLogP, predicted using Data Warrior.^22,23^

f ND = not determined.

The enantiomers had comparable solubilities (134 μg/mL for **44** and 138 μg/mL for **45**), and permeabilities (77.8 nm/s for **44** and 93.6 nm/s for **45**). With CL_int_ < 10.0 μL/min/mg of protein in rats and humans, both compounds could be considered metabolically stable.^38^ Our lead compound, **44** showed levels of activity analogous to those of **4** (pIC_50_ = 8.54), while PFI was reduced (PFI = 7.14 for **44**, compared to 9.08 for **4**). As both compounds contain 5 aromatic rings, this difference in PFI resulted from an advantageous decrease in chrom LogD_7.4_ (2.14 for **44**, compared to 4.08 for **4**), as high lipophilicity has been shown to correlate with attrition through the drug development phases.^39^

Our SAR optimization strategy throughout this work consisted of improving potency and reducing lipophilicity. Plotting pIC_50_ against cLogP for a range of compounds allows a series to be ranked to suggest molecules with improved physicochemical properties. This was carried out for all 16 analogues, with **4** included as a comparator (Figure 7). Compound **44** (LipE = 6.16) demonstrated a significantly higher LipE than **4** (LipE = 3.51).

**Figure 7:**
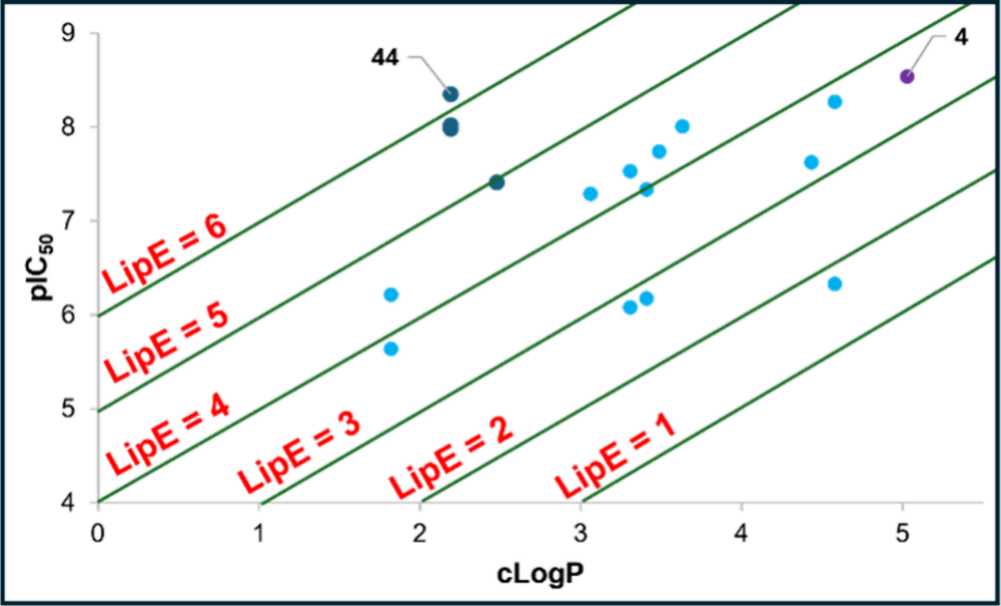
Plot of pIC_50_ versus cLogP for all 16 analogues from the tricyclic scaffold and comparator **4**. Potency against BRD4-BD1, as determined by FRET binding.^21^ cLogP values generated using Data Warrior.^22^ Data points for synthesized isoxazole-containing analogues are shown in cyan and triazole-containing analogues in blue. Data point for literature benchmark **4** shown in purple.

In conclusion, a novel triazinoindole scaffold for targeted BET inhibition was synthesized using a traditional SAR approach. Late-stage functionalization gave a series of *N*-substituted indole derivatives. Preliminary results indicated that many had submicromolar levels of potency but retained the characteristically high PFI of several documented BET inhibitors. The second iteration, implemented primarily to reduce PFI and improve LipE by making strategic structural modifications to the core scaffold, afforded **29** and **30**, both of which had PFIs < 7. However, their progression was hindered by poor membrane permeabilities. Further optimization yielded **40**, which demonstrated nanomolar potency against BRD4-BD1 and had an acceptable PFI of 7.15. **40** was separated to produce enantiomers **44** and **45**, with **44** being more potent. **44** displayed comparable activity against BRD4-BD1 to clinical candidate **4** and possessed lower lipophilicity, as demonstrated by an improvement in LipE. The structurally close homologue (compound **4**) was reported to display pan-inhibitory effects on binding to BRD2, BRD3, and BRD4 (IC_50_ = 0.8, 1.4, and 1.1 nM, respectively) along with potent cytotoxicity, consistent with the role of c-Myc in driving proliferation in a panel of human lung cancer cell lines.^20^ Further work is ongoing in our laboratories to confirm BET-dependent cellular effects for **44**, and these results will be communicated in due course.

## Supplementary Material



## Notes

Synthetic methods for preparation of **5**, **7**–**32** and **34**–**45**; docking of **5a**, **5b**, **44** and **45** in BRD4-BD1 binding pocket (PDF)

## ABBREVIATIONS

BET: bromodomain and extra-terminal domain
BRD: bromodomain-containing protein
BRDT: bromodomain testis-specific protein
BD: bromodomain
Bpin: boronic acid pinacol ester
CAD: charged aerosol detection
chrom: chromatographic
CL_int_: intrinsic clearance
c-Myc: cellular myelocytomatosis
ET: extra-terminal
FP: fluorescence polarization
FRET: fluorescence resonance energy transfer
LipE: lipophilic efficiency
PAMPA: parallel artificial membrane permeability assay
PFI: property forecast index
