# Supplementary material for: Discovery of Highly Potent BET Inhibitors based on a Tractable Tricyclic Scaffold
Source: ACS Med Chem Lett. 2025 Mar 21;16(4):588–95. doi: 10.1021/acsmedchemlett.4c00621 (PMC11995238; doi:10.1021/acsmedchemlett.4c00621)
Supplement: Supplementary file 1 [file ml4c00621_si_001.pdf]

Supporting Information

Discovery of Highly Potent BET Inhibitors based on a Tractable Tricyclic Scaffold

Jaffer M. Zaidi[1], Eleonora Comeo[1], Andrew Baxter[2], Alex G. S. Preston[2], Weng C. Chan[1] and Michael J. Stocks[1]*.

[1]Biodiscovery Institute, School of Pharmacy, University of Nottingham, University Park, Nottingham, NG7 2RD, UK; [2]Medicines Research Centre, GSK, Gunnels Wood Road, Stevenage, SG1 2NY, UK.

*Corresponding Author: michael.stocks@nottingham.ac.uk

**Contents**	**Page**
General Details	S2
Experimental Procedures	S5
Supplementary Figures	S61
References	S65

General Details

No unexpected or unusually high safety hazards were encountered. Unless otherwise stated, all reactions were carried out under an atmosphere of nitrogen in conventional glassware. All water was deionised before use. Chemicals and solvents of analytical and HPLC grade were purchased from commercial suppliers and used without further purification. Anhydrous solvents were transferred from the Sure Seal bottles by syringe under an atmosphere of nitrogen. Room temperature varied between 18 °C and 25 °C. All reported compounds are >95% pure by HPLC.

Analytical TLC was undertaken on Merck aluminium-backed silica gel 60 F_{254} plates. Developed TLC plates were visualised by irradiation with UV light (254 nm). LCMS data was obtained on one of two systems:

- Shimadzu UFLCXR HPLC system coupled to an Applied Biosystems API 2000 LC/MS/MS with ESI, using a Phenomenex Gemini-NX C18 110 Å column (50 mm x 2 mm x 3 μm) at a flow rate of 0.5 mL/min. t_R is reported using a gradient method of 95:5-2:98, A:B over either 5 min (Method 1) or 13 min (Method 2). Solvent A is 0.1% formic acid in H_2O and solvent B is 0.1% formic acid in MeCN
- Waters Acquity UPLC system coupled to a mass spectrometer with ESI (positive and negative modes), using an Acquity UPLC CSH C18 column (50 mm x 2.1 mm x 1.7 μm) at a flow rate of 1.0 mL/min. t_R is reported using a gradient method of 97:3-3:97, A:B over 10 min (Method 3). Solvents A:B are either 0.1% formic acid in H_2O:0.1%

formic acid in MeCN (acidic conditions) or 10 mM aq ammonium bicarbonate:MeCN (basic conditions)

HRMS data was acquired on one of two systems:

- Bruker MicrOTOF II ESI-TOF instrument
- Waters XEVO G2-XS QTOF instrument with ESI (positive mode)

Automated column chromatography was executed on one of two systems:

- Biotage HPFC SP4 system for normal-phase purification, using silica HP (50 µm) cartridges
- Interchim PuriFlash 4100 system for reverse-phase purification, using silica C18-HP (30 µm) cartridges

Chiral separation was achieved by preparative chiral HPLC using a CHIRALPAK IB N-5 column (4.6 mm x 25 cm x 5 µm). NMR spectra were recorded at 298 K on a Bruker AV spectrometer operating at the nominal ^{1}H and ^{13}C frequencies stated. All NMR data was processed using MestReNova. Chemical shifts (δ) are given in ppm and are referenced to residual solvent peaks for ^{1}H and ^{13}C{^{1}H} respectively as appropriate for the NMR solvent used to obtain the spectra. Coupling constants (*J*) are given in Hz. The multiplicity of a ^{1}H or ^{13}C NMR signal is designated by standard abbreviations as defined by ACS Medicinal Chemistry Letters.

All biological evaluation was performed by colleagues at GSK. The *in vitro* assays used to determine potency against BRD4-BD1 (FRET),

hydrophobicity (chrom $LogD_{7.4}$), solubility (CAD), permeability (PAMPA) and metabolic stability (CL_{int}) have all been described previously.[1]

Experimental Procedures

7-(3,5-Dimethylisoxazol-4-yl)-*N*-(2-methoxyethyl)-5*H* [1,2,4]triazino[5,6-*b*]indol-3-amine (**5a**)[2]

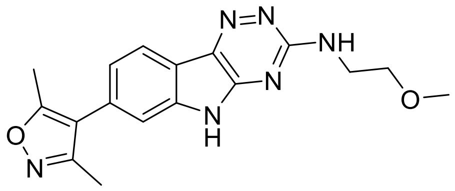

5a

To a solution of **11a** (288 mg, 926 μmol) in NMP (5.0 mL) was added *m*-CPBA (75% pure, 637 mg, 2.77 mmol, 2.99 eq) and the reaction was stirred at rt for 1 h. DIPEA (806 μL, 4.60 mmol, 4.97 eq) and 2-methoxyethylamine (402 μL, 4.60 mmol, 4.97 eq) were then added and the reaction was heated at 110 °C through microwave radiation for 2 h. The reaction mixture was cooled to rt and diluted with EtOAc (100 mL). The organic layer was washed with brine (3 x 100 mL), dried over Na_2SO_4, filtered and concentrated under reduced pressure. The crude material was purified by column chromatography (65:26:9-20:60:20, Cy:EtOAc:IMS) to yield **5a** as a brown solid (138 mg, 44%).

^{1}H NMR (400 MHz, DMSO-d_6) δ: 11.99 (1H, s), 8.15 (1H, d, *J* = 7.9 Hz), 7.34 (1H, s), 7.27 (1H, d, *J* = 7.9 Hz), 3.61-3.49 (4H, m), 3.29 (3H, s), 2.45 (3H, s), 2.27 (3H, s)

$^{13}C\{^{1}H\}$ NMR (101 MHz, DMSO-d_6) δ: 165.7, 159.3, 158.6, 149.7, 139.92, 131.7, 123.7, 121.4, 118.7, 116.7, 112.4, 77.2, 71.4, 58.7, 41.3, 11.7, 10.6.

LCMS (ESI^+): $C_{17}H_{18}N_6O_2$ $[M+H]^+$ m/z calcd 339.2; found 339.2

t_R (Method 2): 3.63 min

8-(3,5-Dimethylisoxazol-4-yl)-*N*-(2-methoxyethyl)-5*H*-[1,2,4]triazino[5,6-*b*]indol-3-amine (**5b**)[2]

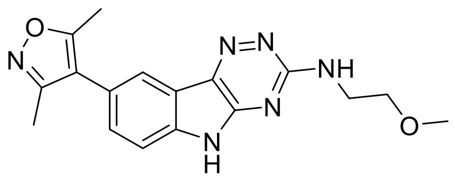

5b

To a solution of **11b** (380 mg, 1.22 mmol) in NMP (6.2 mL) was added *m*-CPBA (70% pure, 902 mg, 3.66 mmol, 3.00 eq) and the reaction was stirred at rt for 1 h. DIPEA (1.06 mL, 6.10 mmol, 5.00 eq) and 2-methoxyethylamine (530 µL, 6.10 mmol, 5.00 eq) were then added and the reaction was heated at 110 °C through microwave radiation for 2 h. The reaction mixture was cooled to rt and diluted with EtOAc (100 mL). The organic layer was washed with brine (3 x 100 mL), dried over Na_2SO_4, filtered and concentrated under reduced pressure. The crude material was purified by column chromatography (88:9:3-40:45:15, Cy:EtOAc:IMS) to yield **5b** as a yellow solid (303 mg, 73%).

^{1}H NMR (400 MHz, DMSO-d_6) δ: 12.03 (1H, s), 8.05 (1H, s), 7.49-7.45 (2H, m), 3.60-3.52 (4H, m), 3.29 (3H, s), 2.44 (3H, s), 2.26 (3H, s)

^{13}C{^{1}H} NMR (101 MHz, DMSO-d_6) δ: 164.9, 158.2, 148.8, 138.4, 129.0, 123.2, 119.8, 119.7, 116.2, 116.1, 112.3, 112.0, 70.3, 58.0, 11.3, 10.5

LCMS (ESI$^+$): $C_{17}H_{18}N_6O_2$ $[M+H]^+$ m/z calcd 339.2; found 339.0

t_R (Method 2): 3.50 min

Other NH signal not observed in ^{1}H NMR. CH_2 signal missing in ^{13}C NMR.

6-Bromospiro[indoline-3,2'-[1,3]dioxolan]-2-one (**7a**)[3]

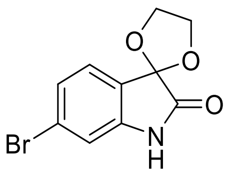

7a

To a solution of 6-bromoisatin (5.00 g, 22.1 mmol) in PhMe (33 mL) was added ethylene glycol (3.71 mL, 66.3 mmol, 3.00 eq) and *p*-TsOH•H_2O (673 mg, 3.54 mmol, 0.160 eq) and the reaction was heated at reflux for 16 h. The reaction mixture was cooled to rt and diluted with EtOAc (30 mL) and H_2O (20 mL). The biphasic mixture was separated and the aqueous phase was then re-extracted with EtOAc (2 x 10 mL). The combined organics were dried over Na_2SO_4, filtered and concentrated under reduced

pressure. The resulting residue was triturated with Et_2O, filtered and dried under vacuum to yield **7a** as a brown solid (2.97 g, 50%).

^{1}H NMR (400 MHz, DMSO-d$_6$) δ: 10.59 (1H, s), 7.28 (1H, d, *J* = 7.9 Hz), 7.21 (1H, dd, *J* = 7.9, 2.0 Hz), 6.99 (1H, d, *J* = 2.0 Hz), 4.38-4.20 (4H, m)

^{13}C{^{1}H} NMR (101 MHz, DMSO-d$_6$) δ: 174.1, 144.4, 126.8, 125.2, 124.4, 123.9, 113.5, 101.2, 65.6

LCMS (ESI$^+$): $C_{10}H_8BrNO_3$ $[M+H]^+$ m/z calcd 270.0; found 270.0

t_R (Method 1): 2.59 min

Spectroscopic data in accordance with literature.[4]

5-Bromospiro[indoline-3,2'-[1,3]dioxolan]-2-one (**7b**)[3]

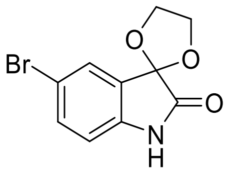

7b

To a solution of 5-bromoisatin (5.00 g, 22.1 mmol) in PhMe (33 mL) was added ethylene glycol (3.71 mL, 66.3 mmol, 3.00 eq) and *p*-TsOH•H_2O (673 mg, 3.54 mmol, 0.160 eq) and the reaction was heated at reflux for 64 h. The reaction mixture was cooled to rt and diluted with EtOAc (30 mL) and H_2O (20 mL). The biphasic mixture was separated and the aqueous

phase was then re-extracted with EtOAc (2 x 10 mL). The combined organics were dried over Na_2SO_4, filtered and concentrated under reduced pressure. The resulting residue was triturated with Et_2O (50 mL), filtered and dried under vacuum to yield **7b** as a brown solid (3.20 g, 54%).

^{1}H NMR (400 MHz, $CDCl_3$) δ: 8.28 (1H, s), 7.46 (1H, d, *J* = 2.1 Hz), 7.43 (1H, dd, *J* = 8.2, 2.1 Hz), 6.73 (1H, d, *J* = 8.2 Hz), 4.60-4.49 (2H, m), 4.37-4.27 (2H, m)

^{13}C{^{1}H} NMR (101 MHz, $CDCl_3$) δ: 175.1, 140.9, 134.6, 128.7, 126.6, 116.0, 112.4, 102.1, 66.2

LCMS (ESI$^+$): $C_{10}H_8BrNO_3$ $[M+H]^+$ m/z calcd 270.0; found 269.9

t_R (Method 1): 2.60 min

NH signal not observed. Spectroscopic data in accordance with literature.[5]

6-(3,5-Dimethylisoxazol-4-yl)spiro[indoline-3,2'-[1,3]dioxolan]-2-one (**8a**)[6]

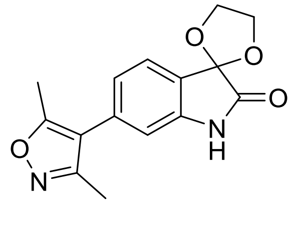

8a

To a microwave vial was added **7a** (1.00 g, 3.70 mmol), 3,5-dimethylisoxazole-4-Bpin (2.06 g, 9.25 mmol, 2.50 eq) and Pd(dppf)Cl_2•DCM (302 mg, 370 µmol, 10 mol%). 2-MeTHF (7.4 mL) was added to the reaction vessel, followed by 2.0 M K_3PO_4 (5.55 mL, 11.1 mmol, 3.00 eq). The reaction was heated at 85 °C through microwave irradiation for 1 h. The reaction mixture was cooled to rt and concentrated under reduced pressure. The crude material was purified first by normal-phase column chromatography (90:10-30:70, Cy:EtOAc) and then by reverse-phase column chromatography (90:10-10:90, H_2O:MeCN) to yield **8a** as an off-white solid (536 mg, 51%).

^{1}H NMR (400 MHz, DMSO-d$_6$) δ: 10.50 (1H, s), 7.41 (1H, d, *J* = 7.6 Hz), 7.01 (1H, dd, *J* = 7.6, 1.7 Hz), 6.79 (1H, d, *J* = 1.7 Hz), 4.41-4.23 (4H, m), 2.41 (3H, s), 2.22 (3H, s)

^{13}C{^{1}H} NMR (101 MHz, DMSO-d$_6$) δ: 174.4, 165.5, 158.0, 143.4, 133.3, 125.3, 123.7, 123.1, 115.7, 110.8, 101.5, 65.5, 11.4, 10.5

LCMS (ESI$^+$): $C_{15}H_{14}N_2O_4$ $[M+H]^+$ m/z calcd 287.1; found 287.0

t_R (Method 1): 2.56 min

5-(3,5-Dimethylisoxazol-4-yl)spiro[indoline-3,2'-[1,3]dioxolan]-2-one (**8b**)[6]

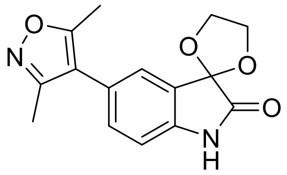

8b

To a microwave vial was added **7b** (2.00 g, 7.41 mmol), 3,5-dimethylisoxazole-4-Bpin (4.12 g, 18.5 mmol, 2.50 eq) and Pd(dppf)Cl_2•DCM (604 mg, 741 µmol, 10 mol%). 2-MeTHF (14.8 mL) was added to the reaction vessel, followed by 2.0 M K_3PO_4 (11.1 mL, 22.2 mmol, 3.00 eq). The reaction was heated at 85 °C through microwave irradiation for 1 h. The reaction mixture was cooled to rt, poured over an ice/water slurry and diluted with EtOAc (100 mL). The biphasic mixture was separated and the aqueous phase was then re-extracted with EtOAc (2 x 100 mL). The combined organics were washed with brine (300 mL), dried over Na_2SO_4, filtered and concentrated under reduced pressure. The crude material was purified first by normal-phase column chromatography (90:10-30:70, Cy:EtOAc) and then by reverse-phase column chromatography (90:10-10:90, H_2O:MeCN) to yield **8b** as a pink solid (1.14 g, 54%).

^{1}H NMR (400 MHz, DMSO-d$_6$) δ: 10.56 (1H, s), 7.32 (2H, dd, *J* = 5.6, 1.9 Hz), 6.96-6.90 (1H, m), 4.39-4.24 (4H, m), 2.36 (1H, s), 2.18 (1H, s)

^{13}C{^{1}H} NMR (101 MHz, DMSO-d$_6$) δ: 174.4, 164.9, 158.1, 142.2, 132.4, 125.42, 125.40, 124.0, 115.5, 110.9, 101.5, 65.5, 11.3, 10.4

LCMS (ESI$^+$): $C_{15}H_{14}N_2O_4$ $[M+H]^+$ m/z calcd 287.1; found 287.0

t_R (Method 2): 4.17 min

Spectroscopic data in accordance with literature.[3]

6-(3,5-Dimethylisoxazol-4-yl)indoline-2,3-dione (**9a**)[3]

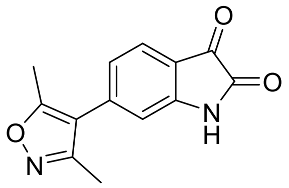

9a

To **8a** (518 mg, 1.81 mmol) was added 12 M HCl (1.06 mL, 12.7 mmol, 7.00 eq) and AcOH (41.6 µL, 724 µmol, 0.400 eq) and the reaction was stirred at rt for 0.25 h. The reaction mixture was poured over an ice/water slurry. The resulting precipitate was collected by filtration, washed with H_2O, EtOH and EtOAc and dried under vacuum to yield **9a** as an orange solid (280 mg, 64%).

^{1}H NMR (400 MHz, DMSO-d_6) δ: 11.08 (1H, s), 7.58 (1H, d, *J* = 7.7 Hz), 7.08 (1H, dd, *J* = 7.7, 1.4 Hz), 6.88 (1H, d, *J* = 1.4 Hz), 2.46 (3H, s), 2.26 (3H, s)

^{13}C{^{1}H} NMR (101 MHz, DMSO-d_6) δ: 183.6, 166.5, 159.5, 157.8, 151.1, 139.8, 125.1, 123.2, 116.8, 115.5, 112.0, 11.7, 10.6

LCMS (ESI$^+$): $C_{13}H_{10}N_2O_3$ $[M+H]^+$ m/z calcd 243.1; found 243.1

t_R (Method 1): 2.48 min

5-(3,5-Dimethylisoxazol-4-yl)indoline-2,3-dione (**9b**)[3]

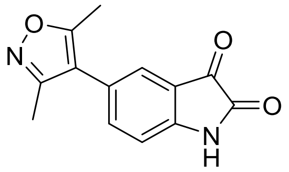

9b

To **8b** (450 mg, 1.57 mmol) was added 12 M HCl (900 µL, 10.8 mmol, 6.88 eq) and AcOH (33.0 µL, 574 µmol, 0.366 eq). The reaction was heated at 90 °C for 1 h. The reaction mixture was cooled to rt and poured over an ice/water slurry. The resulting precipitate was collected by filtration, washed with H_2O, EtOH and EtOAc and dried under vacuum to yield **9b** as an orange solid (242 mg, 64%).

^{1}H NMR (400 MHz, DMSO-d_6) δ: 11.13 (1H, s), 7.58 (1H, dd, J = 8.1, 2.1 Hz), 7.50 (1H, d, J = 2.1 Hz), 7.00 (1H, d, J = 8.1 Hz), 2.37 (3H, s), 2.20 (3H, s)

^{13}C{^{1}H NMR (101 MHz, DMSO-d_6) δ: 184.1, 165.3, 159.4, 158.1, 149.9, 138.7, 124.9, 124.3, 118.3, 114.9, 112.7, 11.3, 10.3

LCMS (ESI$^+$): $C_{13}H_{10}N_2O_3$ $[M+H]^+$ m/z calcd 243.1; found 243.1

t_R (Method 2): 3.86 min

Spectroscopic data in accordance with literature.[3]

7-(3,5-Dimethylisoxazol-4-yl)-5*H*-[1,2,4]triazino[5,6-*b*]indole-3-thiol (**10a**)[7]

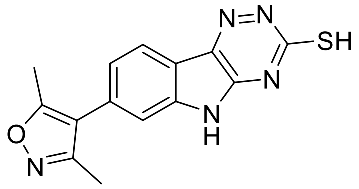

10a

To a suspension of **9a** (265 mg, 1.09 mmol) in H_2O (11 mL) was added thiosemicarbazide (129 mg, 1.42 mmol, 1.30 eq) and K_2CO_3 (452 mg, 3.27 mmol, 3.00 eq) and the reaction was heated at 110 °C through microwave irradiation for 3 h. The reaction mixture was cooled to rt and acidified by dropwise addition of AcOH. The resulting precipitate was filtered, washed with H_2O and dried to yield **10a** as a yellow solid (209 mg, 64%).

^{1}H NMR (400 MHz, DMSO-d$_6$) δ: 8.07 (1H, d, *J* = 8.0 Hz), 7.40 (1H, s), 7.34 (1H, dd, *J* = 8.0, 1.5 Hz), 2.46 (3H, s), 2.27 (3H, s)

^{13}C{^{1}H} NMR (101 MHz, DMSO-d$_6$) δ: 179.1, 165.9, 158.0, 149.5, 143.5, 135.3, 133.4, 123.8, 122.2, 116.9, 115.8, 113.0, 11.5, 10.6

LCMS (ESI$^+$): $C_{14}H_{11}N_5OS$ $[M+H]^+$ m/z calcd 298.1; found 298.1

t_R (Method 1): 2.47 min

NH and SH signals not observed.

8-(3,5-Dimethylisoxazol-4-yl)-5*H*-[1,2,4]triazino[5,6-*b*]indole-3-thiol (**10b**)[7]

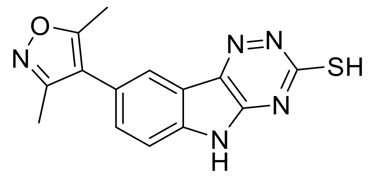

10b

To a suspension of **9b** (150 mg, 619 µmol) in H_2O (6.2 mL) was added thiosemicarbazide (73.4 mg, 805 µmol, 1.30 eq) and K_2CO_3 (257 mg, 1.86 mmol, 3.00 eq) and the reaction was heated at 110 °C through microwave irradiation for 2 h. The reaction mixture was cooled to rt and acidified by dropwise addition of AcOH. The resulting precipitate was filtered, washed with H_2O and dried to yield **10b** as an orange solid (102 mg, 55%).

^{1}H NMR (400 MHz, DMSO-d$_6$) δ: 7.99 (1H, d, *J* = 1.8 Hz), 7.61 (1H, dd, *J* = 8.3, 1.8 Hz), 7.52 (1H, d, *J* = 8.3 Hz), 2.42 (3H, s), 2.24 (3H, s)

^{13}C{^{1}H} NMR (101 MHz, DMSO-d$_6$) δ: 179.2, 165.3, 158.3, 149.5, 142.5, 135.5, 132.6, 124.7, 122.1, 118.4, 115.5, 113.4, 11.3, 10.4

LCMS (ESI$^+$): $C_{14}H_{11}N_5OS$ $[M+H]^+$ m/z calcd 298.1; found 298.1

t$_R$ (Method 1): 2.41 min

NH and SH signals not observed.

3,5-Dimethyl-4-(3-(methylthio)-5*H*-[1,2,4]triazino[5,6-*b*]indol-7-yl)isoxazole (**11a**)[8]

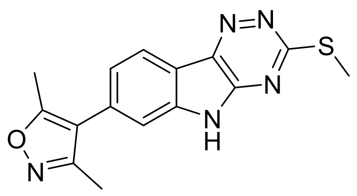

11a

To a suspension of **10a** (189 mg, 636 µmol) in DMF (2.8 mL) was added Et_3N (177 µL, 1.27 mmol, 2.00 eq) and MeI (59.4 µL, 954 µmol, 1.50 eq) and the reaction was stirred at rt for 18 h. The reaction mixture was poured into an ice/water slurry. The resulting precipitate was filtered, washed with H_2O and dried to yield **11a** as a yellow solid (158 mg, 80%).

^{1}H NMR (400 MHz, DMSO-d_6) δ: 12.65 (1H, s), 8.35 (1H, d, *J* = 7.9 Hz), 7.51 (1H, s), 7.45-7.36 (1H, m), 2.66 (3H, s), 2.48 (3H, s), 2.29 (3H, s)

^{13}C{^{1}H} NMR (101 MHz, DMSO-d_6) δ: 167.7, 165.7, 158.1, 147.1, 140.6, 132.3, 123.3, 121.8, 116.9, 116.0, 112.7, 13.4, 11.5, 10.6

LCMS (ESI^{+}): $C_{15}H_{13}N_5OS$ $[M+H]^{+}$ m/z calcd 312.1; found 312.1

t_R (Method 1): 2.70 min

Cq signal missing.

3,5-Dimethyl-4-(3-(methylthio)-5*H*-[1,2,4]triazino[5,6-*b*]indol-8-yl)isoxazole (**11b**)[8]

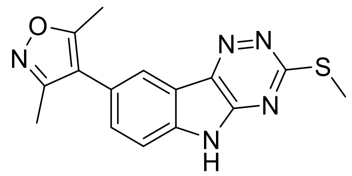

11b

To a suspension of **10b** (87.7 mg, 295 µmol) in DMF (1.3 mL) was added Et_3N (61.6 µL, 442 µmol, 1.50 eq) and MeI (18.4 µL, 295 µmol, 1.00 eq) and the reaction was stirred at rt for 2 h. An additional 0.50 eq of Et_3N and MeI were added and the reaction was stirred for a further 15 h. The reaction mixture was poured into an ice/water slurry. The resulting precipitate was filtered, washed with H_2O and dried to yield **11b** as a yellow solid (65.4 mg, 71%).

^{1}H NMR (400 MHz, DMSO-d$_6$) δ: 12.72 (1H, s), 8.27 (1H, s), 7.66 (2H, d, *J* = 2.4 Hz), 2.66 (3H, s), 2.45 (3H, s), 2.27 (3H, s)

^{13}C{^{1}H} NMR (101 MHz, DMSO-d$_6$) δ: 167.9, 165.2, 158.3, 147.0, 140.6, 139.4, 131.6, 124.1, 121.6, 118.3, 115.8, 113.1, 13.4, 11.3, 10.5

LCMS (ESI$^+$): $C_{15}H_{13}N_5OS$ $[M+H]^+$ m/z calcd 312.1; found 312.1

t_R (Method 1): 2.72 min

5-Benzyl-7-(3,5-dimethylisoxazol-4-yl)-*N*-(2-methoxyethyl)-5*H*-[1,2,4]triazino[5,6-*b*]indol-3-amine (**12a**)[9]

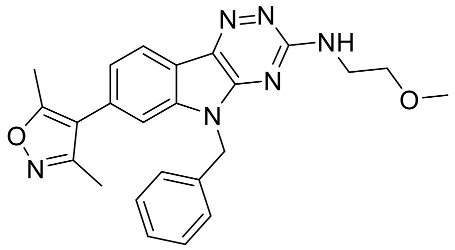

12a

To a solution of **5a** (25.0 mg, 73.9 μmol) in DMF (1.0 mL) was added K_2CO_3 (12.3 mg, 88.7 μmol, 1.20 eq) and the reaction was stirred at rt for 1 h. Benzyl bromide (11.4 μL, 96.0 μmol, 1.30 eq) was added, the reaction was heated to 70 °C and stirred for a further 19 h. The reaction mixture was diluted with H_2O (10 mL) and then extracted with EtOAc (3 x 15 mL). The combined organics were washed with brine (50 mL), dried over Na_2SO_4, filtered and concentrated under reduced pressure. The crude material was

purified by reverse-phase column chromatography (90:10-10:90, H_2O:MeCN) to yield **12a** as a yellow solid (19.9 mg, 63%).

^{1}H NMR (400 MHz, DMSO-d$_6$) δ: 8.19 (1H, d, *J* = 8.0 Hz), 7.55 (1H, s), 7.45-7.21 (6H, m), 5.50 (2H, s), 3.65-3.53 (4H, m), 3.28 (3H, s), 2.37 (3H, s), 2.20 (3H, s)

^{13}C{^{1}H} NMR (101 MHz, DMSO-d$_6$) δ: 165.5, 158.2, 136.4, 128.7, 127.7, 122.9, 120.0, 118.2, 116.1, 111.2, 70.3, 57.9, 43.6, 11.4, 10.4

LCMS (ESI$^+$): $C_{24}H_{24}N_6O_2$ [M+H]$^+$ m/z calcd 429.2; found 429.1

t_R (Method 2): 5.10 min

HRMS (ESI$^+$): $C_{24}H_{24}N_6O_2$ [M+H]$^+$ m/z calcd 429.2034; found 429.2038

NH signal not observed in ^{1}H NMR. Some Cq signals missing in ^{13}C NMR.

5-Benzyl-8-(3,5-dimethylisoxazol-4-yl)-*N*-(2-methoxyethyl)-5*H*-[1,2,4]triazino[5,6-*b*]indol-3-amine (**12b**)[9]

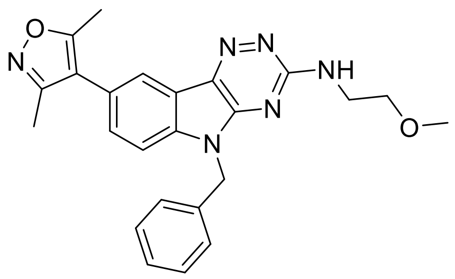

12b

To a solution of **5b** (25.0 mg, 73.9 μmol) in DMF (1.0 mL) was added K_2CO_3 (12.3 mg, 88.7 μmol, 1.20 eq) and the reaction was stirred at rt for 1 h. Benzyl bromide (11.4 μL, 96.0 μmol, 1.30 eq) was added and the reaction was stirred for a further 18 h. The reaction mixture was diluted with H_2O (10 mL) and then extracted with EtOAc (3 x 15 mL). The combined organics were washed with brine (50 mL), dried over Na_2SO_4, filtered and concentrated under reduced pressure. The crude material was purified by reverse-phase column chromatography (90:10-10:90, H_2O:MeCN) to yield **12b** as an orange solid (20.4 mg, 64%).

^{1}H NMR (400 MHz, DMSO-d$_6$) δ: 8.10 (1H, d, *J* = 1.7 Hz), 7.66 (1H, d, *J* = 8.5 Hz), 7.49 (1H, dd, *J* = 8.5, 1.7 Hz), 7.41-7.22 (5H, m), 5.49 (2H, s), 3.65-3.50 (4H, m), 3.27 (3H, s), 2.42 (3H, s), 2.24 (3H, s)

^{13}C{^{1}H} NMR (101 MHz, DMSO-d$_6$) δ: 165.0, 158.3, 148.3, 136.4, 129.0, 128.8, 127.7, 127.5, 123.8, 119.9, 119.6, 115.9, 111.2, 70.2, 57.9, 43.7, 11.3, 10.5

LCMS (ESI$^+$): $C_{24}H_{24}N_6O_2$ $[M+H]^+$ m/z calcd 429.2; found 429.0

t_R (Method 2): 5.11 min

HRMS (ESI$^+$): $C_{24}H_{24}N_6O_2$ $[M+H]^+$ m/z calcd 429.2034; found 429.2033

NH signal not observed in ^{1}H NMR. Some Cq signals missing in ^{13}C NMR.

7-(3,5-Dimethylisoxazol-4-yl)-5-(4-fluorobenzyl)-*N*-(2-methoxyethyl)-5*H*-[1,2,4]triazino[5,6-*b*]indol-3-amine (**13a**)[9]

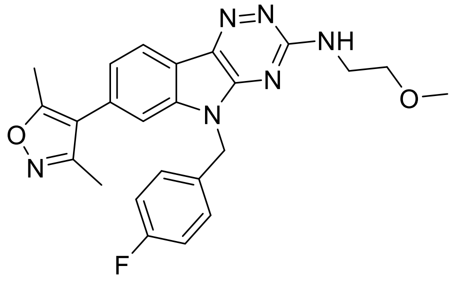

13a

To a solution of **5a** (25.0 mg, 73.9 μmol) in DMF (1.0 mL) was added K_2CO_3 (12.3 mg, 88.7 μmol, 1.20 eq) and the reaction was stirred at rt for 1 h. 4-Fluorobenzyl chloride (11.5 μL, 96.0 μmol, 1.30 eq) was added, the reaction was heated to 70 °C and stirred for a further 17 h. The reaction mixture was cooled to rt, diluted with H_2O (10 mL) and then extracted with EtOAc (3 x 15 mL). The combined organics were washed with brine (50 mL), dried over Na_2SO_4, filtered and concentrated under reduced pressure. The crude material was purified by reverse-phase column chromatography (90:10-10:90, H_2O:MeCN) to yield **13a** as a yellow solid (23.6 mg, 72%).

^{1}H NMR (400 MHz, DMSO-d$_6$) δ: 8.19 (1H, d, *J* = 7.9 Hz), 7.58 (1H, s), 7.51-7.42 (2H, m), 7.32 (1H, dd, *J* = 7.9, 1.5 Hz), 7.14 (2H, t, *J* = 8.9 Hz), 5.49 (2H, s), 3.67-3.53 (4H, m), 3.29 (3H, s), 2.39 (3H, s), 2.21 (3H, s)

$^{13}C\{^{1}H\}$ NMR (101 MHz, DMSO-d$_6$) δ: 165.5, 162.8, 160.4, 158.2, 132.7, 129.9, 123.0, 120.0, 118.2, 116.1, 115.6, 115.4, 111.2, 70.3, 57.9, 42.9, 11.4, 10.4

LCMS (ESI$^+$): $C_{24}H_{23}FN_6O_2$ [M+H]$^+$ m/z calcd 447.2; found 447.0

t$_R$ (Method 2): 5.25 min

NH signal not observed in ^{1}H NMR. Some Cq signals missing in ^{13}C NMR.

8-(3,5-Dimethylisoxazol-4-yl)-5-(4-fluorobenzyl)-*N*-(2-methoxyethyl)-5*H*-[1,2,4]triazino[5,6-*b*]indol-3-amine (**13b**)[9]

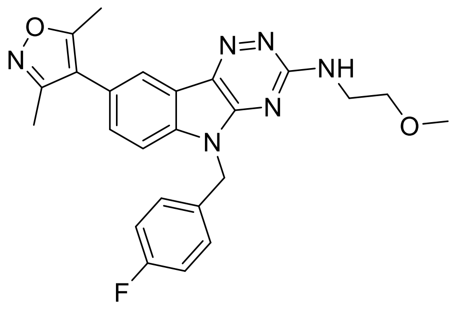

13b

To a solution of **5b** (25.0 mg, 73.9 µmol) in DMF (1.0 mL) was added K_2CO_3 (12.3 mg, 88.7 µmol, 1.20 eq) and the reaction was stirred at rt for 1 h. 4-Fluorobenzyl chloride (11.5 µL, 96.0 µmol, 1.30 eq) was added and the reaction was stirred for 16 h. The reaction was heated to 70 °C and stirred for a further 3 h. The reaction mixture was cooled to rt, diluted with H_2O (10 mL) and then extracted with EtOAc (3 x 15 mL). The combined organics were washed with brine (50 mL), dried over Na_2SO_4, filtered and

concentrated under reduced pressure. The crude material was purified by reverse-phase column chromatography (90:10-10:90, H_2O:MeCN) to yield **13b** as an orange solid (22.2 mg, 67%).

^{1}H NMR (400 MHz, DMSO-d$_6$) δ: 8.10 (1H, d, J = 8.1 Hz), 7.69 (1H, d, J = 8.2 Hz), 7.53-7.40 (3H, m), 7.15 (2H, t, J = 8.8 Hz), 5.48 (2H, s), 3.66-3.52 (4H, m), 3.28 (3H, s), 2.42 (3H, s), 2.25 (3H, s)

^{13}C{^{1}H} NMR (101 MHz, DMSO-d$_6$) δ: 165.1, 162.8, 160.4, 158.3, 132.6, 129.7, 123.9, 119.9, 119.6, 115.9, 115.7, 115.7, 115.5, 111.2, 70.2, 57.9, 43.0, 40.4, 11.3, 10.5

LCMS (ESI$^+$): $C_{24}H_{23}FN_6O_2$ [M+H]$^+$ m/z calcd 447.2; found 447.1

t_R (Method 2): 5.26 min

HRMS (ESI$^+$): $C_{24}H_{23}FN_6O_2$ [M+H]$^+$ m/z calcd 447.1940; found 447.1938

NH signal not observed in ^{1}H NMR. Some Cq signals missing in ^{13}C NMR.

Phenyl(tetrahydro-2*H*-pyran-4-yl)methanone (**14**)[10]

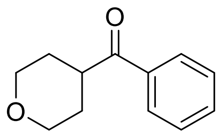

14

To a solution of $TsNHNH_2$ (186 mg, 1.00 mmol) in MeOH (4.0 mL) was added 4-oxo-THP (92.4 µL, 1.00 mmol, 1.00 eq) and the reaction was

stirred at rt for 2 h. The reaction mixture was concentrated under reduced pressure. To the resulting residue was added PhCHO (102 μL, 1.00 mmol, 1.00 eq), Cs_2CO_3 (489 mg, 1.50 mmol, 1.50 eq) and dioxane (8.0 mL) and the reaction was heated at reflux for a further 16 h. The reaction mixture was cooled to rt, quenched with sat aq NH_4Cl (5.0 mL) and extracted with DCM (3 x 10 mL). The combined organics were dried over Na_2SO_4, filtered and concentrated under reduced pressure. The crude material was purified by column chromatography (100:0-50:50, Cy:EtOAc) to yield **14** as a yellow oil (89.9 mg, 47%).

^{1}H NMR (400 MHz, $CDCl_3$) δ: 7.97-7.90 (2H, m), 7.60-7.53 (1H, m), 7.51-7.44 (2H, m), 4.09-4.01 (2H, m), 3.60-3.45 (3H, m), 1.95-1.72 (4H, m)

^{13}C{^{1}H} NMR (101 MHz, $CDCl_3$) δ: 201.9, 135.9, 133.2, 128.9, 128.4, 67.4, 42.7, 29.2

LCMS (ESI$^+$): $C_{12}H_{14}O_2$ $[M+H]^+$ m/z calcd 191.1; found 191.1

t_R (Method 1): 2.63 min

Spectroscopic data in accordance with literature.[11]

Pyridin-2-yl(tetrahydro-2*H*-pyran-4-yl)methanone (**15**)[10]

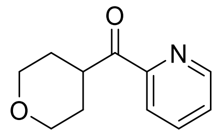

15

To a solution of $TsNHNH_2$ (186 mg, 1.00 mmol) in MeOH (4.0 mL) was added 4-oxo-THP (92.4 µL, 1.00 mmol, 1.00 eq) and the reaction was stirred at rt for 2 h. The reaction mixture was concentrated under reduced pressure. To the resulting residue was added 2-py-CHO (95.1 µL, 1.00 mmol, 1.00 eq), Cs_2CO_3 (489 mg, 1.50 mmol, 1.50 eq) and dioxane (8.0 mL) and the reaction was heated at reflux for a further 41 h. The reaction mixture was cooled to rt, quenched with sat aq NH_4Cl (5.0 mL) and extracted with DCM (3 x 10 mL). The combined organics were dried over Na_2SO_4, filtered and concentrated under reduced pressure. The crude material was purified by column chromatography (100:0-50:50, Cy:EtOAc) to yield **15** as a yellow solid (126 mg, 66%).

^{1}H NMR (400 MHz, $CDCl_3$) δ: 8.67 (1H, d, *J* = 4.8 Hz), 8.03 (1H, d, *J* = 7.7 Hz), 7.84 (1H, td, *J* = 7.7, 1.6 Hz), 7.47 (1H, ddd, *J* = 7.7, 4.8, 1.6 Hz), 4.15-3.99 (3H, m), 3.64-3.55 (2H, m), 1.87-1.75 (4H, m)

^{13}C{^{1}H} NMR (101 MHz, $CDCl_3$) δ: 202.8, 152.5, 148.8, 137.4, 127.3, 122.8, 67.5, 41.5, 28.7

LCMS (ESI$^+$): $C_{11}H_{13}NO_2$ $[M+H]^+$ m/z calcd 192.1; found 192.1

t_R (Method 1): 2.34 min

Spectroscopic data in accordance with literature.[12]

tert-Butyl 4-benzoylpiperidine-1-carboxylate (**16**)[10]

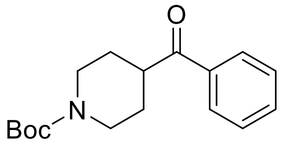

16

To a solution of $TsNHNH_2$ (186 mg, 1.00 mmol) in MeOH (4.0 mL) was added 1-Boc-4-piperidone (199 mg, 1.00 mmol, 1.00 eq) and the reaction was stirred at rt for 2.5 h. The reaction mixture was concentrated under reduced pressure. To the resulting residue was added PhCHO (102 µL, 1.00 mmol, 1.00 eq), Cs_2CO_3 (489 mg, 1.50 mmol, 1.50 eq) and dioxane (8.0 mL) and the reaction was heated at reflux for a further 40 h. The reaction mixture was cooled to rt, quenched with sat aq NH_4Cl (5.0 mL) and extracted with DCM (3 x 10 mL). The combined organics were dried over Na_2SO_4, filtered and concentrated under reduced pressure. The crude material was purified by column chromatography (100:0-60:40, Cy:EtOAc) to yield **16** as a yellow oil (183 mg, 63%).

^{1}H NMR (400 MHz, $CDCl_3$) δ: 7.93 (2H, d, *J* = 7.0 Hz), 7.60-7.53 (1H, m), 7.51-7.44 (2H, m), 4.20-4.12 (2H, m), 3.45-3.35 (1H, m), 2.95-2.85 (2H, m), 1.89-1.80 (2H, m), 1.76-1.63 (2H, m), 1.46 (9H, s)

^{13}C{^{1}H} NMR (101 MHz, $CDCl_3$) δ: 202.2, 154.9, 136.0, 133.2, 128.9, 128.4, 79.8, 43.6, 43.4, 28.6, 28.5

LCMS (ESI$^+$): $C_{17}H_{23}NO_3$ $[M+H]^+$ m/z calcd 290.2; found 190.1

t_R (Method 1): 2.94 min

m/z of Boc deprotected product observed in LCMS. Spectroscopic data in accordance with literature.[11]

tert-Butyl 4-picolinoylpiperidine-1-carboxylate (**17**)[10]

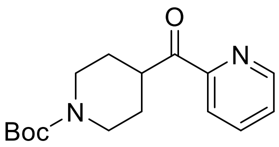

17

To a solution of $TsNHNH_2$ (1.86 g, 10.0 mmol) in MeOH (40 mL) was added 1-Boc-4-piperidone (1.99 g, 10.0 mmol, 1.00 eq) and the reaction was stirred at rt for 2 h. The reaction mixture was concentrated under reduced pressure. To the resulting residue was added 2-py-CHO (951 μL, 10.0 mmol, 1.00 eq), Cs_2CO_3 (4.89 g, 15.0 mmol, 1.50 eq) and dioxane (80 mL) and the reaction was heated at reflux for a further 19 h. The reaction mixture was cooled to rt, quenched with sat aq NH_4Cl (50 mL) and diluted with DCM (100 mL). The biphasic mixture was separated and the aqueous phase was then re-extracted with DCM (2 x 50 mL) The combined organics were dried over Na_2SO_4, filtered and concentrated under reduced pressure. The crude material was purified by column chromatography (80:20-40:60, Cy:EtOAc) to yield **17** as a yellow oil (1.70 g, 59%).

^{1}H NMR (400 MHz, $CDCl_3$) δ: 8.66 (1H, d, *J* = 4.8 Hz), 8.01 (1H, d, *J* = 7.8 Hz), 7.83 (1H, td, *J* = 7.8 Hz), 7.50-7.42 (1H, m), 4.22-4.06 (2H, m), 4.01 (1H, tt, *J* = 11.5, 3.6 Hz), 2.90 (2H, t, *J* = 12.0 Hz), 1.87 (2H, d, *J* = 12.3 Hz), 1.68-1.54 (2H, m), 1.44 (9H, s)

^{13}C{^{1}H} NMR (101 MHz, $CDCl_3$) δ: 203.2, 154.9, 152.5, 148.9, 137.3, 127.3, 122.7, 79.5, 43.4, 42.2, 28.6, 28.0

LCMS (ESI^+): $C_{16}H_{22}N_2O_3$ $[M+H]^+$ m/z calcd 291.2; found 190.8

t_R (Method 1): 2.81 min

m/z of Boc deprotected product observed in LCMS. Spectroscopic data in accordance with literature.[12]

Phenyl(tetrahydro-2*H*-pyran-4-yl)methanol (**18**)[13]

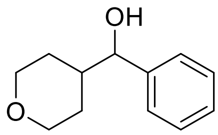

18

To a solution of **14** (74.7 mg, 393 μmol) in MeOH (2.5 mL) at 0 °C was added $NaBH_4$ (44.6 mg, 1.18 mmol, 3.00 eq). The reaction was warmed to rt and stirred for 2 h. The reaction mixture was quenched with sat aq NH_4Cl (5.0 mL) and extracted with DCM (3 x 10 mL). The combined organics were dried over Na_2SO_4, filtered and concentrated under reduced pressure to yield **18** as a colourless oil (67.1 mg, 89%).

^{1}H NMR (400 MHz, DMSO-d$_6$) δ: 7.34-7.26 (4H, m), 7.25-7.19 (1H, m), 5.18 (1H, d, *J* = 4.5 Hz), 4.29-4.20 (1H, m), 3.90-3.71 (2H, m), 3.26-3.08 (2H, m), 1.73-1.61 (2H, m), 1.33-1.17 (3H, m), 1.13-1.04 (1H, m)

^{13}C{^{1}H} NMR (101 MHz, DMSO-d$_6$) δ: 144.4, 127.8, 126.7, 126.6, 76.5, 67.1, 66.8, 42.3, 29.1, 28.7

t_R (Method 1): 2.42 min

OH signal not observed. Correct m/z not observed in LCMS. Spectroscopic data in accordance with literature.[14]

Pyridin-2-yl(tetrahydro-2*H*-pyran-4-yl)methanol (**19**)[13]

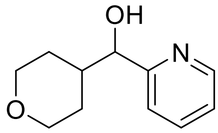

19

To a solution of **15** (104 mg, 544 μmol) in MeOH (3.5 mL) at 0 °C was added $NaBH_4$ (61.7 mg, 1.63 mmol, 3.00 eq). The reaction was warmed to rt and stirred for 2 h. The reaction mixture was quenched with sat aq NH_4Cl (10 mL) and extracted with DCM (3 x 15 mL). The combined organics were dried over Na_2SO_4, filtered and concentrated under reduced pressure to yield **19** as a yellow oil (97.3 mg, 93%).

^{1}H NMR (400 MHz, DMSO-d_6) δ: 8.48 (1H, d, *J* = 4.9 Hz), 7.76 (1H, td, *J* = 7.8, 1.9 Hz), 7.43 (1H, d, *J* = 7.8 Hz), 7.27-7.20 (1H, m), 5.32 (1H, d, *J* = 5.4 Hz), 4.36 (1H, t, *J* = 5.4 Hz), 3.86-3.75 (2H, m), 3.25-3.13 (2H, m), 1.98-1.85 (1H, m), 1.46-1.28 (3H, m), 1.25-1.16 (1H, m)

^{13}C{^{1}H} NMR (101 MHz, DMSO-d_6) δ: 163.3, 148.2, 136.3, 122.0, 120.8, 76.9, 67.0, 66.9, 41.1, 29.2, 27.5

LCMS (ESI$^+$): $C_{11}H_{15}NO_2$ [M+H]$^+$ m/z calcd 194.1; found 194.2

t_R (Method 1): 2.37 min

tert-Butyl 4-(hydroxy(phenyl)methyl)piperidine-1-carboxylate (**20**)[13]

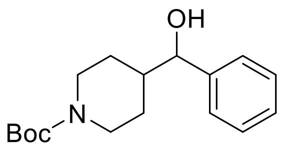

20

To a solution of **16** (163 mg, 563 μmol) in MeOH (3.6 mL) at 0 °C was added $NaBH_4$ (63.9 mg, 1.69 mmol, 3.00 eq). The reaction was warmed to rt and stirred for 2 h. The reaction mixture was quenched with sat aq NH_4Cl (10 mL) and extracted with DCM (3 x 15 mL). The combined organics were dried over Na_2SO_4, filtered and concentrated under reduced pressure to yield **20** as a colourless oil (109 mg, 66%).

^{1}H NMR (400 MHz, $CDCl_3$) δ: 7.35-7.19 (5H, m), 4.31 (1H, d, *J* = 7.5 Hz), 4.16-3.94 (2H, m), 2.68-2.40 (3H, m), 1.98-1.85 (1H, m), 1.78-1.63 (1H, m), 1.41 (9H, s), 1.27-1.17 (2H, m)

^{13}C{^{1}H} NMR (101 MHz, $CDCl_3$) δ: 154.9, 143.2, 128.3, 127.7, 126.6, 79.4, 78.4, 43.8, 43.7, 43.5, 28.5, 28.4, 28.3

LCMS (ESI^{+}): $C_{17}H_{25}NO_3$ $[M+H]^{+}$ m/z calcd 292.2; found 192.1

t_R (Method 1): 1.38 min

OH signal not observed. m/z of Boc deprotected product observed in LCMS. Spectroscopic data in accordance with literature.[15]

tert-Butyl 4-(hydroxy(pyridin-2-yl)methyl)piperidine-1-carboxylate (**21**)[13]

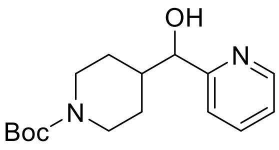

21

To a solution of **17** (1.66 g, 5.72 mmol) in MeOH (37 mL) at 0 °C was added $NaBH_4$ (649 mg, 17.2 mmol, 3.00 eq). The reaction was warmed to rt and stirred for 2 h. The reaction mixture was quenched with sat aq NH_4Cl (40 mL) and diluted with DCM (100 mL). The biphasic mixture was separated and the aqueous layer was then re-extracted with DCM (2 x 50 mL). The combined organics were dried over Na_2SO_4, filtered and

concentrated under reduced pressure to yield **21** as a yellow oil (1.65 g, 99%).

^{1}H NMR (400 MHz, DMSO-d_6) δ: 8.48 (1H, d, *J* = 5.9 Hz), 7.76 (1H, td, *J* = 7.8, 1.8 Hz), 7.43 (1H, d, *J* = 7.8 Hz), 7.27-7.18 (1H, m), 5.33 (1H, d, *J* = 5.1 Hz), 4.39 (1H, t, *J* = 5.3 Hz), 3.92 (2H, d, *J* = 11.2 Hz), 2.60-2.56 (3H, m), 1.93-1.81 (1H, m), 1.37 (9H, s), 1.28-1.08 (2H, m)

^{13}C{^{1}H} NMR (101 MHz, DMSO-d_6) δ: 163.3, 153.8, 148.2, 136.3, 122.0, 120.8, 78.4, 76.6, 41.8, 28.4, 28.1, 26.3

LCMS (ESI$^+$): $C_{16}H_{24}N_2O_3$ $[M+H]^+$ m/z calcd 293.2; found 193.1

t_R (Method 1): 2.26 min

OH signal not observed. m/z of Boc deprotected product observed in LCMS. Spectroscopic data in accordance with literature.[16]

7-(3,5-Dimethylisoxazol-4-yl)-*N*-(2-methoxyethyl)-5-(phenyl(tetrahydro-2*H*-pyran-4-yl)methyl)-5*H*-[1,2,4]triazino[5,6-*b*]indol-3-amine (**22**)[6]

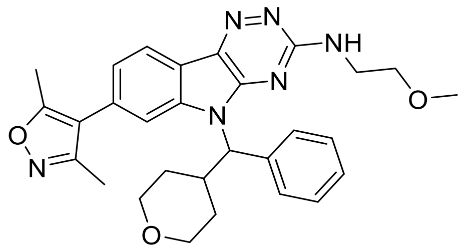

22

To a solution of **5a** (16.5 mg, 48.8 µmol) in PhMe (1.0 mL) was added **18** (18.7 mg, 97.6 µmol, 2.00 eq), PPh_3 (51.2 mg, 195 µmol, 4.00 eq) and DIAD (38.3 µL, 195 µmol, 4.00 eq) and the reaction was stirred at rt for 18 h. The reaction mixture was concentrated under reduced pressure. The crude material was purified by column chromatography (100:0-0:100, DCM:EtOAc) to yield **22** as an orange oil (19.4 mg, 78%).

^{1}H NMR (400 MHz, DMSO-d_6) δ: 8.16 (1H, d, *J* = 7.9 Hz), 7.94 (1H, s), 7.77 (2H, d, *J* = 7.5 Hz), 7.38-7.23 (4H, m), 5.58 (1H, d, *J* = 11.2 Hz), 3.91-3.77 (2H, m), 3.71-3.61 (4H, m), 3.61-3.50 (2H, m), 3.34 (3H, s), 2.46 (3H, s), 2.30 (3H, s), 1.43-1.19 (5H, m)
^{13}C{^{1}H} NMR (101 MHz, DMSO-d_6) δ: 165.7, 158.3, 128.7, 128.6, 128.1, 123.4, 120.4, 117.8, 116.1, 111.8, 70.2, 65.5, 58.1, 54.9, 30.6, 30.1, 11.5, 10.5
LCMS (ESI$^+$): $C_{29}H_{32}N_6O_3$ $[M+H]^+$ m/z calcd 513.3; found 513.2
t_R (Method 2): 5.21 min

NH signal not observed in ^{1}H NMR. Some Cq signals missing in ^{13}C NMR.

8-(3,5-Dimethylisoxazol-4-yl)-*N*-(2-methoxyethyl)-5-(phenyl(tetrahydro-2*H*-pyran-4-yl)methyl)-5*H*-[1,2,4]triazino[5,6-*b*]indol-3-amine (**23**)[6]

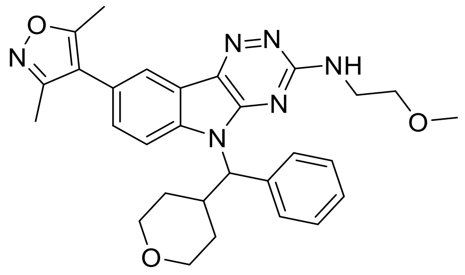

23

To a solution of **5b** (19.1 mg, 56.5 μmol) in PhMe (2.0 mL) was added **18** (32.6 mg, 170 μmol, 3.00 eq), PPh_3 (74.2 mg, 283 μmol, 5.00 eq) and DIAD (55.6 μL, 283 μmol, 5.00 eq) and the reaction was stirred at rt for 3 h. The reaction mixture was concentrated under reduced pressure. The crude material was purified by column chromatography (50:50-0:100, DCM:EtOAc) to yield **23** as an orange oil (16.2 mg, 56%).

^{1}H NMR (400 MHz, DMSO-d_6) δ: 8.08-8.00 (2H, m), 7.77 (2H, d, *J* = 7.1 Hz), 7.52 (1H, dd, *J* = 8.5, 1.9 Hz), 7.38-7.22 (3H, m), 5.52 (1H, d, *J* = 11.1 Hz), 3.93-3.76 (4H, m), 3.68-3.57 (4H, m), 3.33 (3H, s), 2.42 (3H, s), 2.25 (3H, s), 1.44-1.23 (5H, m)

^{13}C{^{1}H} NMR (101 MHz, DMSO-d_6) δ: 165.1, 158.3, 148.1, 139.0, 138.5, 128.7, 128.5, 128.1, 123.9, 119.7, 119.5, 115.8, 111.6, 70.3, 66.6, 59.8, 58.1, 30.6, 30.2, 11.3, 10.5

LCMS (ESI$^+$): $C_{29}H_{32}N_6O_3$ $[M+H]^+$ m/z calcd 513.3; found 512.9

t_R (Method 2): 5.08 min

NH signal not observed in ^{1}H NMR. Some Cq signals missing in ^{13}C NMR.

7-(3,5-Dimethylisoxazol-4-yl)-*N*-(2-methoxyethyl)-5-(pyridin-2-yl(tetrahydro-2*H*-pyran-4-yl)methyl)-5*H*-[1,2,4]triazino[5,6-*b*]indol-3-amine (**24**)[6]

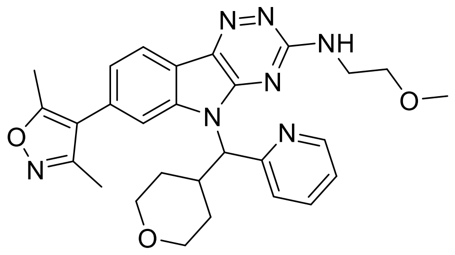

24

To a solution of **5a** (21.5 mg, 63.6 μmol) in PhMe (1.3 mL) was added **19** (24.6 mg, 127 μmol, 2.00 eq), PPh_3 (66.9 mg, 255 μmol, 4.00 eq) and DIAD (50.1 μL, 255 μmol, 4.00 eq) and the reaction was stirred at rt for 5 h. The reaction mixture was concentrated under reduced pressure. The crude material was purified by column chromatography (50:50-0:100, DCM:EtOAc) to yield **24** as a yellow oil (8.90 mg, 27%).

^{1}H NMR (400 MHz, DMSO-d_6) δ: 8.57 (1H, d, *J* = 3.8 Hz), 8.16 (1H, d, *J* = 8.0 Hz), 7.87-7.74 (2H, m), 7.60 (1H, d, *J* = 8.0 Hz), 7.35-7.27 (2H, m), 5.78 (1H, d, *J* = 11.2 Hz), 3.91-3.72 (4H, m), 3.70-3.57 (4H, m), 3.32 (3H,

s), 2.43 (3H, s), 2.26 (3H, s), 1.63-1.50 (1H, m), 1.46-1.33 (1H, m), 1.31-1.20 (2H, m), 1.20-1.04 (1H, m)

$^{13}C\{^1H\}$ NMR (101 MHz, DMSO-d_6) δ: 165.5, 158.2, 149.3, 137.3, 124.1, 123.4, 122.9, 122.0, 120.8, 119.8, 118.4, 116.2, 112.4, 76.9, 70.2, 67.0, 66.4, 58.0, 54.9, 30.8, 29.4, 27.5, 11.5, 10.6

LCMS (ESI^+): $C_{28}H_{31}N_7O_3$ $[M+H]^+$ m/z calcd 514.3; found 514.0

t_R (Method 2): 4.64 min

HRMS (ESI^+): $C_{28}H_{31}N_7O_3$ $[M+H]^+$ m/z calcd 514.2562; found 514.2564

NH signal not observed in ^{1}H NMR. Some Cq signals missing in ^{13}C NMR.

tert-Butyl 4-((7-(3,5-dimethylisoxazol-4-yl)-3-((2-methoxyethyl)amino)-5*H*-[1,2,4]triazino[5,6-*b*]indol-5-yl)(phenyl)methyl)piperidine-1-carboxylate (**25**)[6]

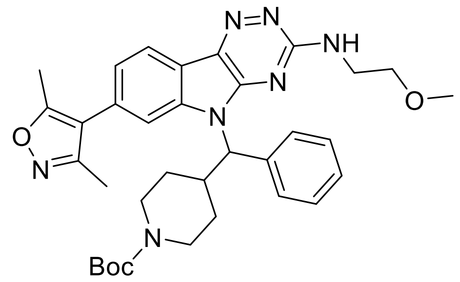

25

To a solution of **5a** (19.4 mg, 57.3 µmol) in PhMe (2.0 mL) was added **20** (50.1 mg, 172 µmol, 3.00 eq), PPh_3 (75.2 mg, 287 µmol, 5.00 eq) and DIAD (56.3 µL, 287 µmol, 5.00 eq) and the reaction was stirred at rt for

22 h. The reaction mixture was concentrated under reduced pressure. The crude material was purified by column chromatography (50:50-0:100, DCM:EtOAc) to yield **25** as an orange oil (28.4 mg, 81%).

^{1}H NMR (400 MHz, DMSO-d$_6$) δ: 8.15 (1H, d, *J* = 7.6 Hz), 7.90 (1H, s), 7.76 (1H, d, *J* = 7.6 Hz), 7.37-7.22 (5H, m), 5.57 (1H, d, *J* = 10.9 Hz), 4.06-3.83 (2H, m), 3.69-3.59 (4H, m), 3.59-3.48 (2H, m), 3.33 (3H, s), 2.45 (3H, s), 2.29 (3H, s), 1.46-1.43 (1H, m), 1.35 (9H, s), 1.24-1.05 (4H, m)

^{13}C{^{1}H} NMR (101 MHz, DMSO-d$_6$) δ: 165.5, 158.3, 153.7, 133.2, 132.2, 132.0, 131.5, 131.4, 128.8, 128.7, 128.5, 128.1, 123.0, 119.8, 118.2, 116.3, 111.5, 78.5, 70.4, 59.8, 58.1, 28.0, 21.6, 20.8, 14.1, 11.5, 10.5

LCMS (ESI$^+$): $C_{34}H_{41}N_7O_4$ [M+H]$^+$ m/z calcd 612.3; found 612.1

t_R (Method 1): 2.97 min

NH signal not observed in ^{1}H NMR. Some Cq signals missing in ^{13}C NMR.

tert-Butyl 4-((7-(3,5-dimethylisoxazol-4-yl)-3-((2-methoxyethyl)amino)-5*H*-[1,2,4]triazino[5,6-*b*]indol-5-yl)(pyridin-2-yl)methyl)piperidine-1-carboxylate (**26**)[6]

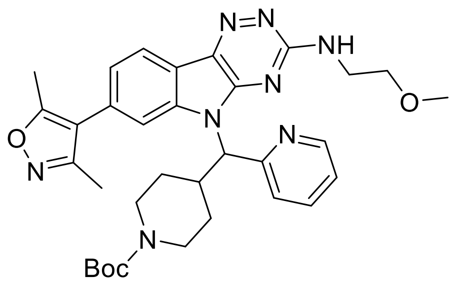

26

To a solution of **5a** (30.0 mg, 88.7 μmol) in PhMe (4.0 mL) was added **21** (61.0 mg, 209 μmol, 2.30 eq), PPh_3 (93.1 mg, 355 μmol, 4.00 eq) and DIAD (69.7 μL, 355 μmol, 4.00 eq) and the reaction was stirred at rt for 3 h. The reaction mixture was concentrated under reduced pressure. The crude material was purified by column chromatography (100:0-0:100, DCM:EtOAc) to yield **26** as a yellow solid (10.3 mg, 19%).

LCMS (ESI^+): $C_{33}H_{40}N_8O_4$ $[M+H]^+$ m/z calcd 613.3; found 613.1

t_R (Method 1): 2.88 min

7-(3,5-Dimethylisoxazol-4-yl)-*N*-(2-methoxyethyl)-5-(phenyl(piperidin-4-yl)methyl)-5*H*-[1,2,4]triazino[5,6-*b*]indol-3-amine (**27**)[17]

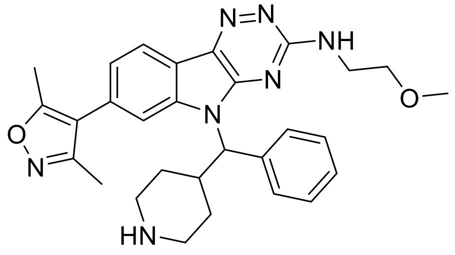

27

To **25** (28.4 mg, 46.4 µmol) was added 4.0 M HCl in dioxane (1.0 mL) and the reaction was stirred at rt for 2 h. The reaction mixture was concentrated under reduced pressure. The crude material was purified by reverse-phase column chromatography (90:10-10:90, H_2O:MeCN) to yield **27** as a yellow oil (8.90 mg, 35%).

^{1}H NMR (400 MHz, DMSO-d_6) δ: 8.89 (1H, s), 8.16 (1H, d, *J* = 7.8 Hz), 7.88 (1H, s), 7.76 (1H, d, *J* = 7.8 Hz), 7.40-7.24 (5H, m), 5.62 (1H, d, *J* = 11.7 Hz), 3.70-3.58 (4H, m), 3.33 (3H, s), 3.25-3.16 (1H, m), 2.96-2.70 (3H, m), 2.44 (3H, s), 2.27 (3H, s), 1.72-1.34 (5H, m)

^{13}C{^{1}H} NMR (101 MHz, DMSO-d_6) δ: 165.5, 158.3, 128.8, 128.4, 128.2, 123.1, 119.8, 118.3, 116.3, 111.7, 70.4, 58.1, 42.8, 42.6, 40.4, 26.1, 11.5, 10.5

LCMS (ESI$^+$): $C_{29}H_{33}N_7O_2$ $[M+H]^+$ m/z calcd 512.3; found 512.2

t_R (Method 2): 4.06 min

Other NH signal not observed in ^{1}H NMR. Some Cq signals missing in ^{13}C NMR.

7-(3,5-Dimethylisoxazol-4-yl)-*N*-(2-methoxyethyl)-5-(piperidin-4-yl(pyridin-2-yl)methyl)-5*H*-[1,2,4]triazino[5,6-*b*]indol-3-amine (**28**)[17]

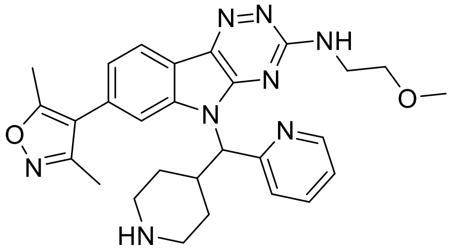

28

To **26** (10.0 mg, 16.3 µmol) was added 4.0 M HCl in dioxane (1.0 mL) and the reaction was stirred at rt for 0.25 h. The reaction mixture was concentrated under reduced pressure. The crude material was purified by reverse-phase column chromatography (90:10-10:90, H_2O:MeCN) to yield **28** as a yellow oil (6.80 mg, 81%).

¹H NMR (400 MHz, DMSO-d_6) δ: 8.60 (1H, d, *J* = 3.8 Hz), 8.17 (1H, d, *J* = 8.1 Hz), 7.86-7.72 (2H, m), 7.54 (1H, d, *J* = 7.8 Hz), 7.41-7.26 (2H, m), 5.82 (1H, d, *J* = 11.2 Hz), 3.68-3.57 (4H, m), 3.32 (3H, s), 3.29-3.23 (1H, m), 3.20-3.10 (1H, m), 3.03-2.89 (1H, m), 2.89-2.76 (1H, m), 2.42 (3H, s), 2.25 (3H, s), 1.92-1.77 (1H, m), 1.65-1.30 (3H, m)

LCMS (ESI$^+$): $C_{28}H_{32}N_8O_2$ $[M+H]^+$ m/z calcd 513.3; found 513.2

t_R (Method 2): 3.73 min

2-((7-(3,5-Dimethylisoxazol-4-yl)-5-(piperidin-4-yl(pyridin-2-yl)methyl)-5*H*-[1,2,4]triazino[5,6-*b*]indol-3-yl)amino)ethan-1-ol (**29**)[2, 17]

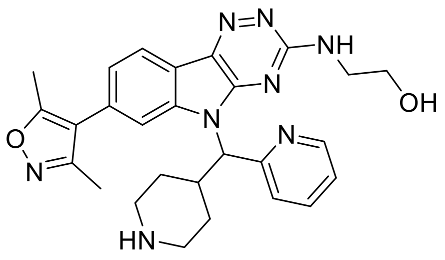

29

To a solution of **31** (173 mg, 295 μmol) in NMP (1.5 mL) was added *m*-CPBA (70% pure, 218 mg, 885 μmol, 3.00 eq) and the reaction was stirred at rt for 1 h. DIPEA (258 μL, 1.48 mmol, 5.00 eq) and ethanolamine (89.5 μL, 1.48 mmol, 5.00 eq) were then added and the reaction was heated at 180 °C through microwave irradiation for 2 h. The reaction mixture was cooled to rt and diluted with EtOAc (15 mL). The organic layer was washed with brine (3 x 75 mL), dried over Na_2SO_4, filtered and concentrated under reduced pressure. The crude material was purified by reverse-phase column chromatography (80:20-0:100, H_2O:MeCN). To the resulting intermediate was added 4.0 M HCl in dioxane (1.7 mL) and the reaction was stirred at rt for 3 h. The reaction mixture was concentrated under reduced pressure. The crude material was purified first by reverse-phase column chromatography (90:10-10:90, H_2O:MeCN)

and then by normal-phase chromatography (100:0-0:100, DCM:MeOH) to yield **29** as a yellow oil (6.60 mg, 4%).

^{1}H NMR (400 MHz, DMSO-d$_6$) δ: 8.59 (1H, d, J = 5.0 Hz), 8.16 (1H, d, J = 8.1 Hz), 7.85-7.76 (2H, m), 7.57 (1H, d, J = 7.8 Hz), 7.41-7.25 (2H, m), 5.81 (1H, d, J = 11.4 Hz), 4.87 (2H, t, J = 5.9 Hz), 3.65 (2H, t, J = 5.9 Hz), 3.28-3.08 (2H, m), 3.00-2.75 (2H, m), 2.42 (3H, s), 2.25 (3H, s), 1.85-1.77 (1H, m), 1.67-1.32 (4H, m)

^{13}C{^{1}H} NMR (101 MHz, DMSO-d$_6$) δ: 165.5, 158.2, 149.3, 137.6, 124.3, 123.5, 123.0, 119.8, 118.5, 116.2, 59.5, 42.4, 26.8, 25.5, 11.6, 10.6

LCMS (ESI$^+$): $C_{27}H_{30}N_8O_2$ [M+H]$^+$ m/z calcd 499.3; found 499.2

t_R (Method 2): 3.37 min

HRMS (ESI$^+$): $C_{27}H_{30}N_8O_2$ [M+H]$^+$ m/z calcd 499.2565; found 499.2568

NH and OH signals not observed in ^{1}H NMR. Some Cq signals missing in ^{13}C NMR.

7-(1,4-Dimethyl-1*H*-1,2,3-triazol-5-yl)-*N*-(2-methoxyethyl)-5-(piperidin-4-yl(pyridin-2-yl)methyl)-5*H*-[1,2,4]triazino[5,6-*b*]indol-3-amine (**30**)[6, 18]

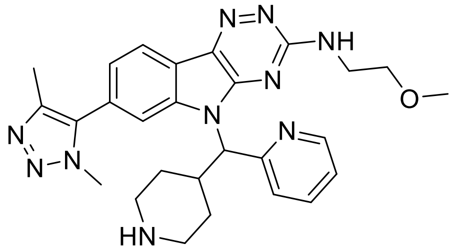

30

To a solution of **39** (28.6 mg, 84.5 µmol) in PhMe (1.3 mL) was added **21** (74.3 mg, 254 µmol, 3.00 eq), PPh_3 (111 mg, 423 µmol, 5.00 eq) and DIAD (83.0 µL, 423 µmol, 5.00 eq) and the reaction was stirred at rt for 20 h. The reaction mixture was concentrated under reduced pressure. The crude material was purified first by normal-phase column chromatography (100:0-0:100, DCM:EtOAc followed by MeOH flush) and then by reverse-phase column chromatography (80:20-0:100, H_2O:MeCN). To the resulting intermediate was added a solution of TFA (200 µL) in DCM (1.0 mL) and the reaction was stirred at rt for 2 h. The reaction mixture was concentrated under reduced pressure. The crude material was purified by reverse-phase column chromatography (90:10-10:90, H_2O:MeCN) to yield **30** as a green oil (2.70 mg, 6%).

^{1}H NMR (400 MHz, DMSO-d$_6$) δ: 8.60 (1H, d, *J* = 4.5 Hz), 8.25 (1H, d, *J* = 8.1 Hz), 7.90 (1H, s), 7.81 (1H, td, *J* = 7.7, 1.8 Hz), 7.54 (1H, d, *J* =

7.9 Hz), 7.43 (1H, d, *J* = 7.9 Hz), 7.39-7.32 (1H, m), 5.84 (1H, d, *J* = 9.9 Hz), 3.96 (3H, s), 3.67-3.57 (4H, m), 3.26 (3H, s), 3.02-2.90 (2H, m), 2.89-2.75 (2H, m), 2.23 (3H, s), 1.90-1.79 (1H, m), 1.56-1.29 (4H, m)

LCMS (ESI$^+$): $C_{27}H_{32}N_{10}O$ $[M+H]^+$ m/z calcd 513.3; found 513.2

t_R (Method 2): 3.28 min

NH signals not observed in ^{1}H NMR. ^{13}C NMR data not obtained due to insufficient material.

tert-Butyl 4-((7-(3,5-dimethylisoxazol-4-yl)-3-(methylthio)-5*H*-[1,2,4]triazino[5,6-*b*]indol-5-yl)(pyridin-2-yl)methyl)piperidine-1-carboxylate (**31**)[6]

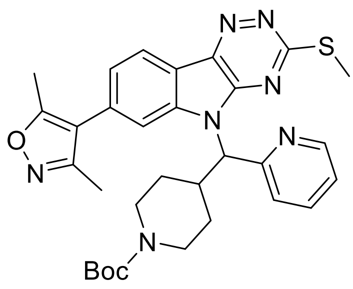

31

To a solution of **11a** (143 mg, 459 µmol) in PhMe (6.7 mL) was added **21** (409 mg, 1.40 mmol, 3.05 eq), PPh_3 (603 mg, 2.30 mmol, 5.00 eq) and DIAD (452 µL, 2.30 mmol, 5.00 eq) and the reaction was stirred at rt for 18 h. The reaction mixture was concentrated under reduced pressure. The

crude material was purified by reverse-phase column chromatography (80:20-0:100, H_2O:MeCN) to yield **31** as a yellow solid (192 mg, 71%).

^{1}H NMR (400 MHz, DMSO-d$_6$) δ: 8.57 (1H, s), 8.38 (1H, d, *J* = 7.9 Hz), 8.03 (1H, s), 7.79 (1H, t, *J* = 7.8 Hz), 7.63 (1H, d, *J* = 7.6 Hz), 7.46 (1H, d, *J* = 8.1 Hz), 7.31 (1H, t, *J* = 6.5 Hz), 5.94 (1H, d, *J* = 11.0 Hz), 4.04-3.76 (2H, m), 3.55-3.40 (2H, m), 2.72 (3H, s), 2.46 (3H, s), 2.29 (3H, s), 1.71-1.54 (1H, m), 1.35 (9H, s), 1.18-1.03 (4H, m)

^{13}C{^{1}H} NMR (101 MHz, DMSO-d$_6$) δ: 168.1, 165.9, 158.1, 155.9, 153.7, 149.4, 146.9, 140.0, 137.3, 132.2, 124.3, 123.7, 123.5, 121.7, 116.9, 116.0, 113.2, 78.5, 35.1, 29.6, 28.5, 28.0, 13.6, 11.5, 10.6

LCMS (ESI$^+$): $C_{31}H_{35}N_7O_3S$ [M+H]$^+$ m/z calcd 586.3; found 586.1

t_R (Method 1): 3.04 min

Some Cq signals missing.

1,4-Dimethyl-5-(tributylstannyl)-1*H*-1,2,3-triazole (**32**)[19]

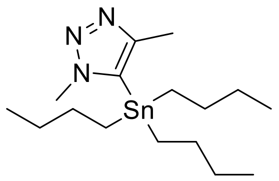

32

To a solution of **34** (900 mg, 9.27 mmol) in THF (19 mL), cooled to -78 °C, was added 2.5 M *n*-BuLi (4.45 mL, 11.1 mmol, 1.20 eq) and the reaction was stirred for 1 h. Bu_3SnCl (3.02 mL, 11.1 mmol, 1.20 eq) was added, the reaction was warmed to rt and stirred for a further 1 h. The reaction mixture was quenched with sat aq NH_4Cl (50 mL) and then extracted with EtOAc (3 x 100 mL). The combined organics were washed with brine (300 mL), dried over Na_2SO_4, filtered and concentrated under reduced pressure. The crude material was purified by column chromatography (80:20-20:80, Cy:EtOAc) to yield **32** as a colourless oil (2.98 g, 83%).

^{1}H NMR (400 MHz, $CDCl_3$) δ: 4.02 (3H, s), 2.34 (3H, s), 1.56-1.45 (6H, m), 1.39-1.26 (6H, m), 1.21-1.14 (6H, m), 0.95-0.84 (9H, m)

^{13}C{^{1}H} NMR (101 MHz, $CDCl_3$) δ: 151.5, 131.8, 38.1, 29.0, 27.3, 17.7, 13.7, 10.2

LCMS (ESI$^+$): $C_{16}H_{33}N_3Sn$ $[M+H]^+$ m/z calcd 388.2; found 388.1

t_R (Method 1): 3.19 min

Spectroscopic data in accordance with literature.[20]

1,4-Dimethyl-1*H*-1,2,3-triazole (**34**)[21]

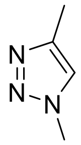

34

To a solution of 1,1-dimethoxyacetone (1.02 mL, 8.47 mmol) in MeOH (20 mL) was added $TsNHNH_2$ (1.58 g, 8.47 mmol, 1.00 eq) and the reaction was stirred at rt for 0.1 h. Et_3N (1.30 mL, 9.32 mmol, 1.10 eq) and $MeNH_2$ (40% pure, 807 µL, 9.32 mmol, 1.10 eq) were added and the reaction was heated at 140 °C through microwave irradiation for 0.1 h. The reaction mixture was cooled to rt and diluted with DCM (150 mL) and H_2O (150 mL). The biphasic mixture was separated and the aqueous layer was then re-extracted with DCM (2 x 150 mL). The combined organics were washed with brine (150 mL), dried over Na_2SO_4, filtered and concentrated under reduced pressure to yield **34** as an orange oil (731 mg, 89%).

^{1}H NMR (400 MHz, DMSO-d_6) δ: 7.75 (1H, s), 3.97 (3H, s), 2.21 (3H, s)
^{13}C{^{1}H} NMR (101 MHz, DMSO-d_6) δ: 141.9, 123.0, 36.0, 10.4

Molecular weight of compound below minimum threshold for LCMS.

6-(1,4-Dimethyl-1*H*-1,2,3-triazol-5-yl)spiro[indoline-3,2'-[1,3]dioxolan]-2-one (**35**)[6]

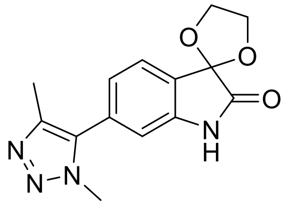

35

To a solution of **7a** (150 mg, 555 µmol) in DMF (1.4 mL) was added **32** (236 mg, 611 µmol, 1.10 eq), $Pd(PPh_3)_4$ (41.7 mg, 36.1 µmol, 6.5 mol%), CuI (15.9 mg, 83.3 µmol, 0.150 eq) and Et_3N (155 µL, 1.10 mmol, 2.00 eq) and the reaction was heated at 95 °C for 2 h. The reaction mixture was cooled to rt and diluted with MeCN (50 mL) and hexane (50 mL). The biphasic mixture was separated and the MeCN layer was concentrated under reduced pressure. The crude material was purified by column chromatography (80:20-20:100, Cy:EtOAc) to yield **35** as a red oil (111 mg, 70%).

^{1}H NMR (400 MHz, DMSO-d$_6$) δ: 10.61 (1H, s), 7.49 (1H, d, *J* = 7.6 Hz), 7.12 (1H, d, *J* = 7.6 Hz), 6.89 (1H, s), 4.42-4.22 (4H, m), 3.93 (3H, s), 2.23 (3H, s)

^{13}C{^{1}H} NMR (101 MHz, DMSO-d$_6$) δ: 174.2, 143.4, 140.3, 133.6, 130.2, 125.5, 125.3, 123.6, 111.0, 101.3, 65.6, 35.5, 10.6

LCMS (ESI$^+$): $C_{14}H_{14}N_4O_3$ $[M+H]^+$ m/z calcd 287.1; found 287.0

t_R (Method 2): 3.36 min

HRMS (ESI$^+$): $C_{14}H_{14}N_4O_3$ $[M+H]^+$ m/z calcd 287.1139; found 287.1136

6-(1,4-Dimethyl-1*H*-1,2,3-triazol-5-yl)indoline-2,3-dione (**36**)[3]

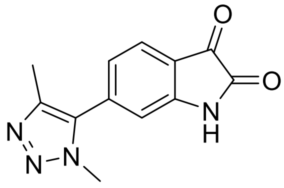

36

To **35** (95.0 mg, 332 µmol) was added 12 M HCl (249 µL, 2.99 mmol, 9.00 eq) and AcOH (19.1 µL, 332 µmol, 1.00 eq) and the reaction was stirred at rt for 4 h. The reaction mixture was diluted with EtOAc (10 mL) and H_2O (10 mL). The resulting biphasic mixture was separated and the aqueous layer was then re-extracted with EtOAc (2 x 10 mL). The combined organics were washed with brine (30 mL), dried over Na_2SO_4, filtered and concentrated under reduced pressure to yield **36** as an orange solid (31.6 mg, 39%).

^{1}H NMR (400 MHz, DMSO-d_6) δ: 11.18 (1H, s), 7.66 (1H, d, *J* = 7.8 Hz), 7.19 (1H, d, *J* = 7.8 Hz), 6.98 (1H, s), 3.97 (3H, s), 2.26 (3H, s)

^{13}C{^{1}H} NMR (101 MHz, DMSO-d_6) δ: 183.7, 159.4, 150.9, 140.8, 136.1, 133.1, 125.1, 123.6, 117.9, 112.4, 35.8, 10.7

LCMS (ESI$^+$): $C_{12}H_{10}N_4O_2$ [M+H]$^+$ m/z calcd 243.1; found 243.1

t_R (Method 2): 2.84 min

HRMS (ESI$^+$): $C_{12}H_{10}N_4O_2$ [M+H]$^+$ m/z calcd 243.0877; found 243.0878

7-(1,4-Dimethyl-1*H*-1,2,3-triazol-5-yl)-5*H*-[1,2,4]triazino[5,6-*b*]indole-3-thiol (**37**)[7]

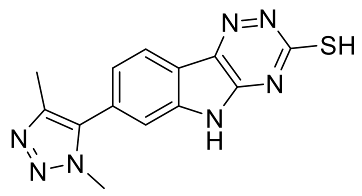

37

To a suspension of **36** (190 mg, 784 µmol) in H_2O (7.9 mL) was added thiosemicarbazide (93.0 mg, 1.02 mmol, 1.30 eq) and K_2CO_3 (325 mg, 2.35 mmol, 3.00 eq) and the reaction was heated at 110 °C through microwave irradiation for 2 h. The reaction mixture was cooled to rt and acidified by dropwise addition of AcOH. The resulting precipitate was filtered, washed with H_2O and dried to yield **37** as a brown solid (151 mg, 65%).

^{1}H NMR (400 MHz, DMSO-d$_6$) δ: 8.15 (1H, d, *J* = 7.9 Hz), 7.52 (1H, s), 7.44 (1H, d, *J* = 7.9 Hz), 3.98 (3H, s), 2.27 (3H, s)

^{13}C{^{1}H} NMR (101 MHz, DMSO-d$_6$) δ: 179.3, 149.7, 143.5, 140.5, 135.2, 133.6, 130.0, 124.0, 122.3, 118.2, 113.5, 35.6, 10.7

LCMS (ESI$^+$): $C_{13}H_{11}N_7S$ $[M+H]^+$ m/z calcd 298.1; found 298.1

t_R (Method 1): 2.21 min

NH and SH signals not observed.

7-(1,4-Dimethyl-1*H*-1,2,3-triazol-5-yl)-3-(methylthio)-5*H*-[1,2,4]triazino[5,6-*b*]indole (**38**)[8]

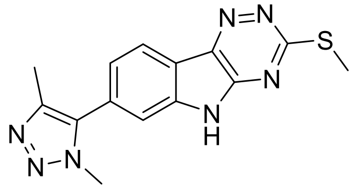

38

To a suspension of **37** (132 mg, 444 μmol) in DMF (6.0 mL) was added Et_3N (124 μL, 868 μmol, 2.00 eq) and MeI (41.5 μL, 666 μmol, 1.50 eq) and the reaction was stirred at rt for 16 h. The reaction mixture was poured into an ice/water slurry. The resulting precipitate was filtered, washed with H_2O and dried to yield **38** as a brown solid (101 mg, 73%).

^{1}H NMR (400 MHz, DMSO-d_6) δ: 8.43 (1H, d, *J* = 8.0 Hz), 7.63 (1H, s), 7.51 (1H, d, *J* = 8.0 Hz), 4.00 (3H, s), 2.66 (3H, s), 2.29 (3H, s)

^{13}C{^{1}H} NMR (101 MHz, DMSO-d_6) δ: 168.1, 147.2, 140.44, 140.40, 140.3, 133.8, 129.0, 123.4, 121.9, 118.1, 113.3, 35.6, 13.4, 10.7

LCMS (ESI$^+$): $C_{14}H_{13}N_7S$ [M+H]$^+$ m/z calcd 312.1; found 312.1

t_R (Method 1): 2.51 min

NH signal not observed.

7-(1,4-Dimethyl-1*H*-1,2,3-triazol-5-yl)-*N*-(2-methoxyethyl)-5*H*-[1,2,4]triazino[5,6-*b*]indol-3-amine (**39**)[2]

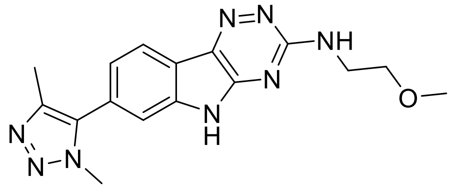

39

To a solution of **38** (85.9 mg, 276 µmol) in NMP (1.4 mL) was added *m*-CPBA (70% pure, 204 mg, 827 µmol, 3.00 eq) and the reaction was stirred at rt for 1 h. DIPEA (240 µL, 1.38 mmol, 5.00 eq) and 2-methoxyethylamine (120 µL, 1.38 mmol, 5.00 eq) were then added and the reaction was heated at 110 °C through microwave irradiation for 2 h. The reaction mixture was cooled to rt and diluted with EtOAc (25 mL). The organic layer was washed with brine (3 x 100 mL), dried over Na_2SO_4, filtered and concentrated under reduced pressure to yield **39** as a brown solid (28.6 mg, 31%).

^{1}H NMR (400 MHz, DMSO-d_6) δ: 8.23 (1H, d, *J* = 8.1 Hz), 7.46 (1H, s), 7.38 (1H, d, *J* = 8.1 Hz), 3.97 (3H, s), 3.62-3.52 (4H, m), 3.29 (3H, s), 2.27 (3H, s)

LCMS (ESI$^+$): $C_{16}H_{18}N_8O$ $[M+H]^+$ m/z calcd 339.2; found 339.1

t_R (Method 1): 2.19 min

NH signals not observed in ^{1}H NMR. ^{13}C NMR data not obtained due to insufficient material.

2-((7-(1,4-Dimethyl-1*H*-1,2,3-triazol-5-yl)-5-(pyridin-2-yl(tetrahydro-2*H*-pyran-4-yl)methyl)-5*H*-[1,2,4]triazino[5,6-*b*]indol-3-yl)amino)ethan-1-ol (**40**)[2, 19]

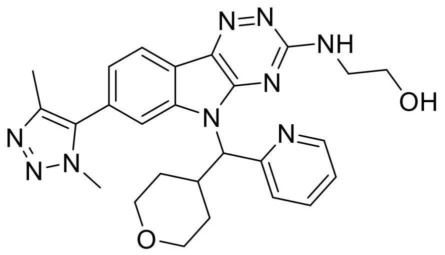

40

To a solution of **43** (336 mg, 714 μmol) in NMP (3.7 mL) was added *m*-CPBA (70% pure, 528 mg, 2.14 mmol, 3.00 eq) and the reaction was stirred at rt for 1 h. DIPEA (622 μL, 3.57 mmol, 5.00 eq) and ethanolamine (216 μL, 1.38 mmol, 5.00 eq) were then added and the reaction was heated at 180 °C through microwave irradiation for 1 h. The reaction mixture was cooled to rt and diluted with EtOAc (40 mL). The organic layer was washed with brine (3 x 200 mL), dried over Na2SO4, filtered and concentrated under reduced pressure. The crude material was purified by reverse-phase column chromatography (90:10-10:90, H_2O:MeCN). To a solution of the resulting intermediate in DMF (1.8 mL) was added **32** (480 mg, 1.24 mmol, 1.74 eq), $Pd(PPh_3)_4$ (53.6 mg, 46.4 μmol, 6.5 mol%), CuI (20.4 mg,

107 μmol, 0.150 eq) and Et_3N (199 μL, 1.43 mmol, 2.00 eq) and the reaction was heated at 95 °C for 1 h. The reaction mixture was cooled to rt and diluted with MeCN (50 mL) and hexane (50 mL). The biphasic mixture was separated and the MeCN layer was concentrated under reduced pressure. The crude material was purified by reverse-phase column chromatography (90:10-10:90, H_2O:MeCN) to yield **40** as an orange solid (78.5 mg, 22%).

^{1}H NMR (400 MHz, DMSO-d$_6$) δ: 8.57 (1H, d, *J* = 4.9 Hz), 8.23 (1H, d, *J* = 8.0 Hz), 7.97 (1H, s), 7.80 (1H, t, *J* = 7.8 Hz), 7.64 (1H, d, *J* = 7.8 Hz), 7.41 (1H, d, *J* = 8.0 Hz), 7.36-7.28 (1H, m), 5.79 (1H, d, *J* = 10.4 Hz), 4.79 (1H, t), 3.97 (3H, s), 3.89-3.72 (2H, m), 3.71-3.62 (4H, m), 3.62-3.45 (1H, m), 3.44-3.36 (1H, m), 3.29-3.23 (1H, m), 2.25 (3H, s), 1.61-1.46 (1H, m), 1.45-1.35 (1H, m), 1.31-1.10 (2H, m)

^{13}C{^{1}H} NMR (101 MHz, DMSO-d$_6$) δ: 156.2, 154.6, 149.3, 137.3, 134.2, 128.6, 124.1, 123.8, 123.5, 123.4, 123.1, 121.2, 119.8, 119.6, 118.5, 113.0, 66.5, 59.5, 35.6, 33.9, 33.4, 30.7, 29.4, 10.7

LCMS (ESI$^+$): $C_{26}H_{29}N_9O_2$ $[M+H]^+$ m/z calcd 500.3; found 500.1

t_R (Method 2): 3.49 min

NH signal not observed.

7-Bromo-5*H*-[1,2,4]triazino[5,6-*b*]indole-3-thiol (**41**)[22]

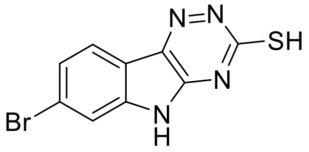

41

To a suspension of 6-bromoisatin (5.00 g, 22.1 mmol) in H_2O (100 mL) was added thiosemicarbazide (2.02 g, 22.1 mmol, 1.00 eq) and K_2CO_3 (4.59 g, 33.2 mmol, 1.50 eq) and the reaction was heated at reflux for 16 h. The reaction mixture was cooled to rt and acidified by dropwise addition of AcOH. The resulting precipitate was filtered and dried to yield **41** as an orange solid (5.61 g, 90%).

^{1}H NMR (400 MHz, DMSO-d_6) δ: 7.84 (1H, d, *J* = 8.1 Hz), 7.55 (1H, d, *J* = 1.7 Hz), 7.35 (1H, dd, *J* = 8.1, 1.7 Hz)

NH and SH signals not observed. Spectroscopic data in accordance with literature.[22]

7-Bromo-3-(methylthio)-5*H*-[1,2,4]triazino[5,6-*b*]indole (**42**)[8]

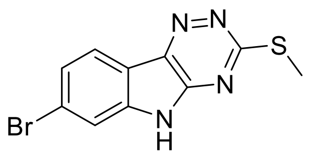

42

To a suspension of **41** (5.50 g, 19.6 mmol) in DMF (20 mL) was added Et_3N (2.73 mL, 19.6 mmol, 1.00 eq) and MeI (1.22 mL, 19.6 mmol, 1.00 eq) and the reaction was stirred at rt for 20 h. The reaction mixture was poured into an ice/water slurry. The resulting precipitate was filtered, washed with H_2O and dried to yield **42** as a gold solid (4.22 g, 73%).

^{1}H NMR (400 MHz, DMSO-d_6) δ: 8.23 (1H, d, *J* = 8.3 Hz), 7.72 (1H, s), 7.58 (1H, d, *J* = 8.3 Hz), 2.65 (3H, s)

^{13}C{^{1}H} NMR (101 MHz, DMSO-d_6) δ: 168.1, 147.0, 141.1, 140.3, 125.4, 123.4, 123.1, 117.0, 115.4, 13.4

LCMS (ESI$^+$): $C_{10}H_7BrN_4S$ $[M+H]^+$ m/z calcd 297.0; found 297.0

t_R (Method 1): 2.72 min

NH signal not observed.

7-Bromo-3-(methylthio)-5-(pyridin-2-yl(tetrahydro-2*H*-pyran-4-yl)methyl)-5*H*-[1,2,4]triazino[5,6-*b*]indole (**43**)[6]

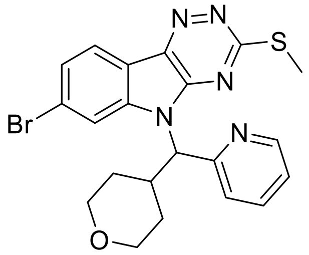

43

To a solution of **42** (500 mg, 1.70 mmol) in PhMe (35 mL) was added **19** (759 mg, 3.93 mmol, 2.31 eq), PPh_3 (2.22 g, 8.48 mmol, 5.00 eq) and DIAD (1.66 mL, 8.48 mmol, 5.00 eq) and the reaction was stirred at rt for 17 h. The reaction mixture was concentrated under reduced pressure. The crude material was purified by reverse-phase column chromatography (90:10-10:90, H_2O:MeCN) to yield **43** as a gold solid (370 mg, 46%).

^{1}H NMR (400 MHz, DMSO-d$_6$) δ: 8.57 (1H, d, *J* = 5.6 Hz), 8.37 (1H, s), 8.23 (1H, d, *J* = 8.3 Hz), 7.80 (1H, t, *J* = 7.7 Hz), 7.62 (2H, dd, *J* = 20.7, 7.7 Hz), 7.35-7.26 (1H, m), 5.89 (1H, d, *J* = 11.1 Hz), 3.92-3.71 (2H, m), 3.59-3.43 (1H, m), 3.44-3.34 (1H, m), 3.31-3.20 (1H, m), 2.70 (3H, s), 1.56-1.44 (2H, m), 1.33-1.27 (1H, m), 1.18-1.10 (1H, m)

^{13}C{^{1}H} NMR (101 MHz, DMSO-d$_6$) δ: 168.4, 155.4, 149.6, 149.3, 146.7, 139.6, 137.3, 126.0, 124.2, 123.62, 123.55, 122.9, 117.0, 115.7, 72.2, 66.5, 66.4, 34.3, 30.3, 29.3, 13.6

LCMS (ESI$^+$): $C_{21}H_{20}BrN_5OS$ [M+H]$^+$ m/z calcd 472.1; found 471.8

t_R (Method 1): 3.06 min

Chiral Separation Procedure: Racemate **40** was purified by chiral HPLC (50:50, 0.2% IPA in heptane:0.2% IPA in EtOH) to yield a separated mixture of enantiomers.

rel-(*R*)-2-((7-(1,4-Dimethyl-1*H*-1,2,3-triazol-5-yl)-5-(pyridin-2-yl(tetrahydro-2*H*-pyran-4-yl)methyl)-5*H*-[1,2,4]triazino[5,6-*b*]indol-3-yl)amino)ethan-1-ol (**44**)

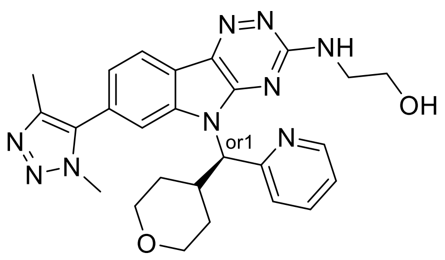

44

44 was synthesised by following the general chiral separation procedure given above and isolated as an orange solid (13.0 mg, 93%).

^{1}H NMR (600 MHz, DMSO-d_6) δ: 8.57 (1H, d, *J* = 4.2 Hz), 8.22 (1H, d, *J* = 7.9 Hz), 7.97 (1H, s), 7.80 (1H, td, *J* = 7.7, 1.8 Hz), 7.63 (1H, d, *J* = 8.0 Hz), 7.41 (1H, dd, *J* = 7.9, 1.5 Hz), 7.34-7.29 (1H, m), 5.79 (1H, d, *J* = 11.1 Hz), 4.78 (1H, t, *J* = 5.6 Hz), 3.97 (3H, s,), 3.87-3.82 (1H, m),

3.78-3.73 (1H, m), 3.67 (2H, q, J = 6.1 Hz), 3.60-3.47 (4H, m), 2.25 (3H, s), 1.61-1.48 (1H, m), 1.46-1.34 (1H, m), 1.32-1.09 (3H, m)

^{13}C{^{1}H} NMR (151 MHz, DMSO-d$_6$) δ: 156.2, 149.3, 137.3, 134.1, 124.1, 123.4, 123.0, 119.8, 119.6, 113.0, 66.5, 59.5, 35.6, 33.9, 30.7, 29.4, 10.7

LCMS (ESI$^+$): $C_{24}H_{24}N_8O$ [M+H]$^+$ m/z calcd 500.25; found 500.25

t$_R$ (Method 3): 2.82 min

NH signal missing in ^{1}H NMR. Some signals missing in ^{13}C NMR.

rel-(*R*)-2-((7-(1,4-Dimethyl-1*H*-1,2,3-triazol-5-yl)-5-(pyridin-2-yl(tetrahydro-2*H*-pyran-4-yl)methyl)-5*H*-[1,2,4]triazino[5,6-*b*]indol-3-yl)amino)ethan-1-ol (**45**)

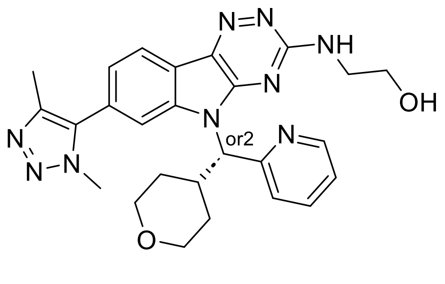

45

45 was synthesised by following the general chiral separation procedure given above and isolated as a yellow solid (11.0 mg, 79%).

^{1}H NMR (600 MHz, DMSO-d$_6$) δ: 8.57 (1H, d, J = 4.2 Hz), 8.23 (1H, d, J = 7.9 Hz), 7.97 (1H, s), 7.80 (1H, td, J = 7.7, 1.8 Hz), 7.63 (1H, d, J =

7.5 Hz), 7.41 (1H, dd, *J* = 7.9, 1.4 Hz), 7.34-7.28 (1H, m), 5.79 (1H, d, *J* = 11.0 Hz), 4.78 (1H, t, *J* = 5.6 Hz), 3.97 (3H, s), 3.89-3.81 (1H, m), 3.79-3.73 (1H, m), 3.67 (2H, q, *J* = 6.2 Hz), 3.63-3.46 (4H, m), 2.25 (3H, s), 1.60-1.49 (1H, m), 1.45-1.33 (1H, m), 1.32-1.11 (3H, m)

$^{13}C\{^{1}H\}$s NMR (151 MHz, DMSO-d_6) δ: 156.2, 149.3, 137.3, 134.1, 124.1, 123.4, 123.0, 119.8, 119.6, 113.0, 66.5, 59.5, 35.6, 33.9, 30.7, 29.4, 10.7

LCMS (ESI^+): $C_{24}H_{24}N_8O$ $[M+H]^+$ m/z calcd 500.25; found 500.25

t_R (Method 3): 2.81 min

HRMS (ESI^+): $C_{24}H_{24}N_8O$ $[M+H]^+$ m/z calcd 500.2517; found 500.2521

NH signal missing in ^{1}H NMR. Some signals missing in ^{13}C NMR.

Supplementary Figures

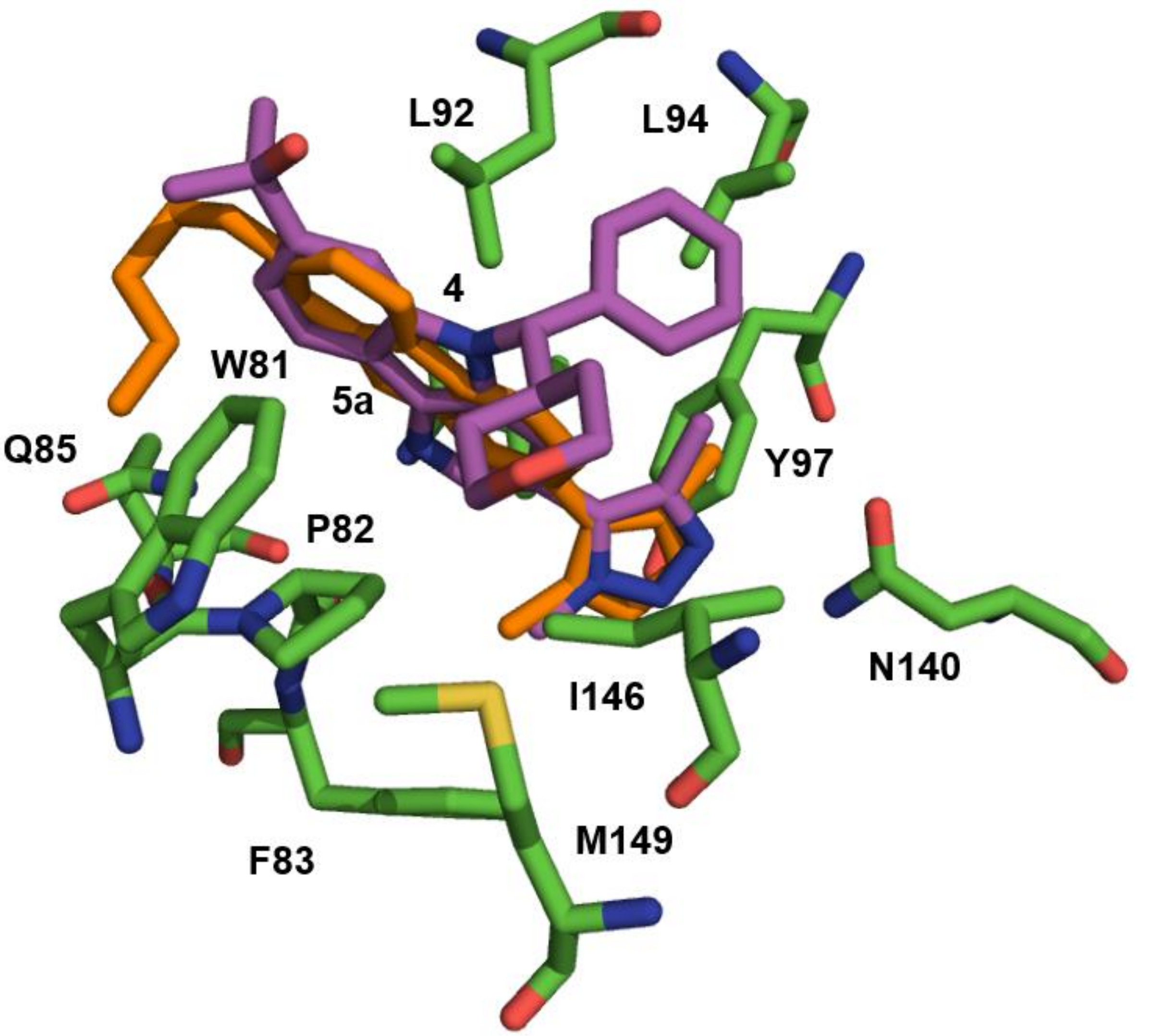

Figure S1. Overlay of literature BET inhibitor **4** and intermediate **5a** in BD1 of BRD4. Amino acids (green), **4** (magenta) and **5a** (orange) are displayed as stick models. Oxygen atoms are shown in red and nitrogen in blue. Key interaction occurs between either triazole (**4**) or isoxazole (**5a**) and asparagine residue in the *N*-acetylated lysine binding pocket. The branched phenyl-THP substituent in **4** interacts with the WPF shelf. Figure generated in PyMOL using PDB 5S9R.[6]

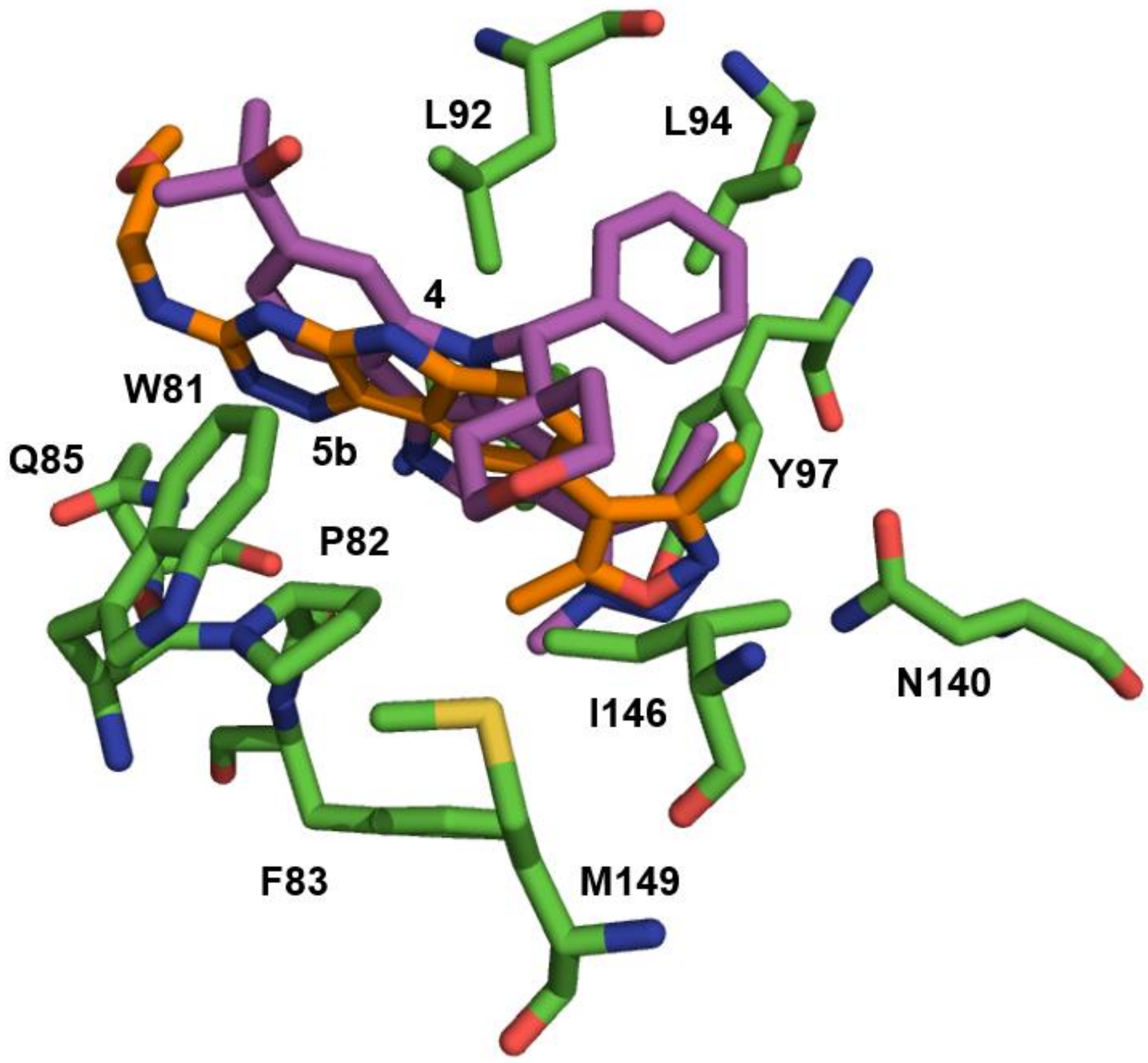

Figure S2. Overlay of literature BET inhibitor **4** and intermediate **5b** in BD1 of BRD4. Amino acids (green), **4** (magenta) and **5b** (orange) are displayed as stick models. Oxygen atoms are shown in red and nitrogen in blue. Key interaction occurs between either triazole (**4**) or isoxazole (**5b**) and asparagine residue in the *N*-acetylated lysine binding pocket. The branched phenyl-THP substituent in **4** interacts with the WPF shelf. Figure generated in PyMOL using PDB 5S9R.[6]

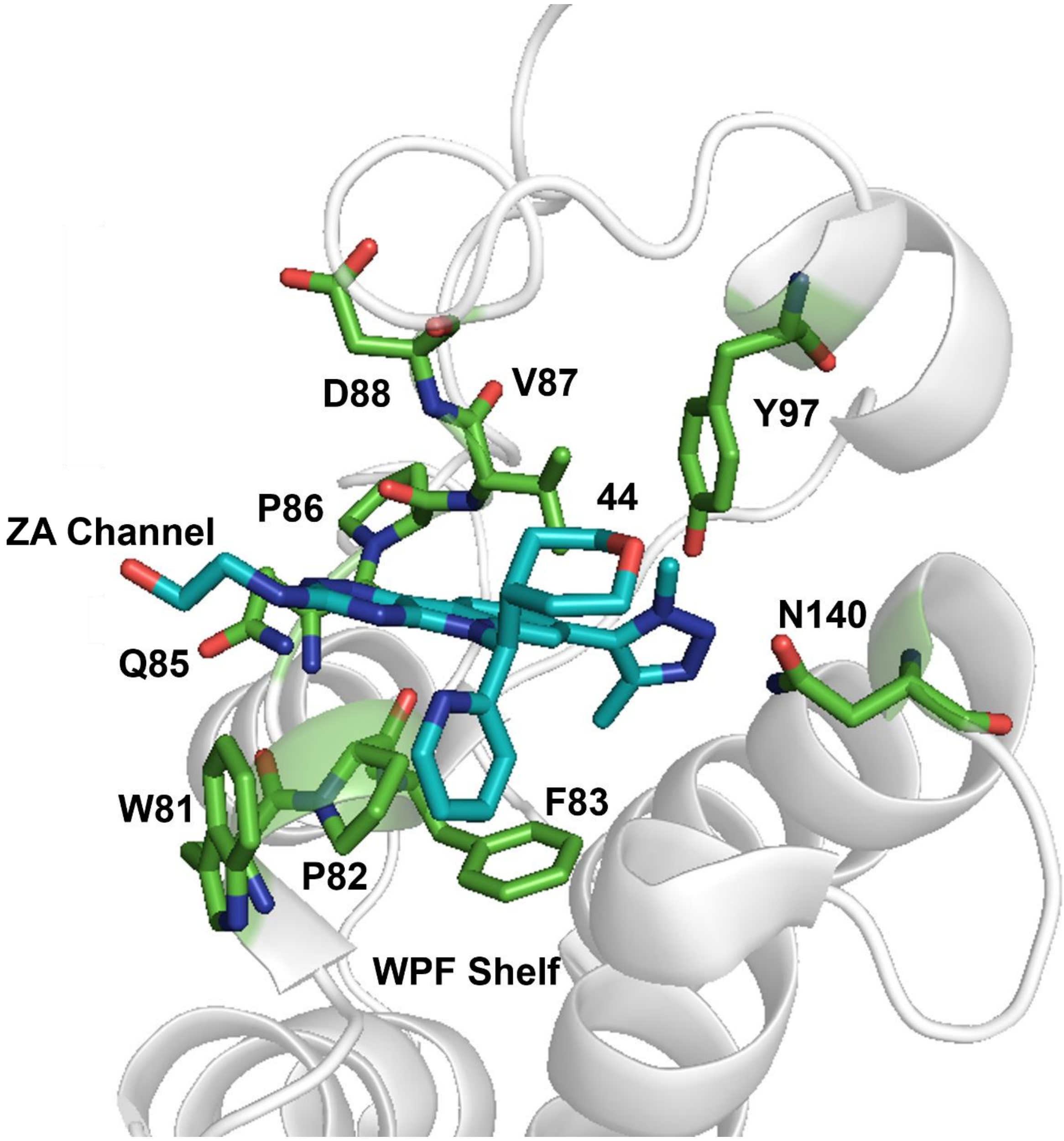

Figure S3. Binding mode of **44** in BD1 of BRD4. The receptor is shown in cartoon representation (grey), while amino acids (green) and **44** (teal) are displayed as stick models. Oxygen atoms are shown in red and nitrogen in blue. Key interaction occurs between triazole and asparagine residue in the *N*-acetylated lysine binding pocket. The triazole, pyridine and triazine moieties in **44** interact with the asparagine residue, WPF shelf and ZA channel respectively. Figure generated in PyMOL using PDB 3ZYU.[23]

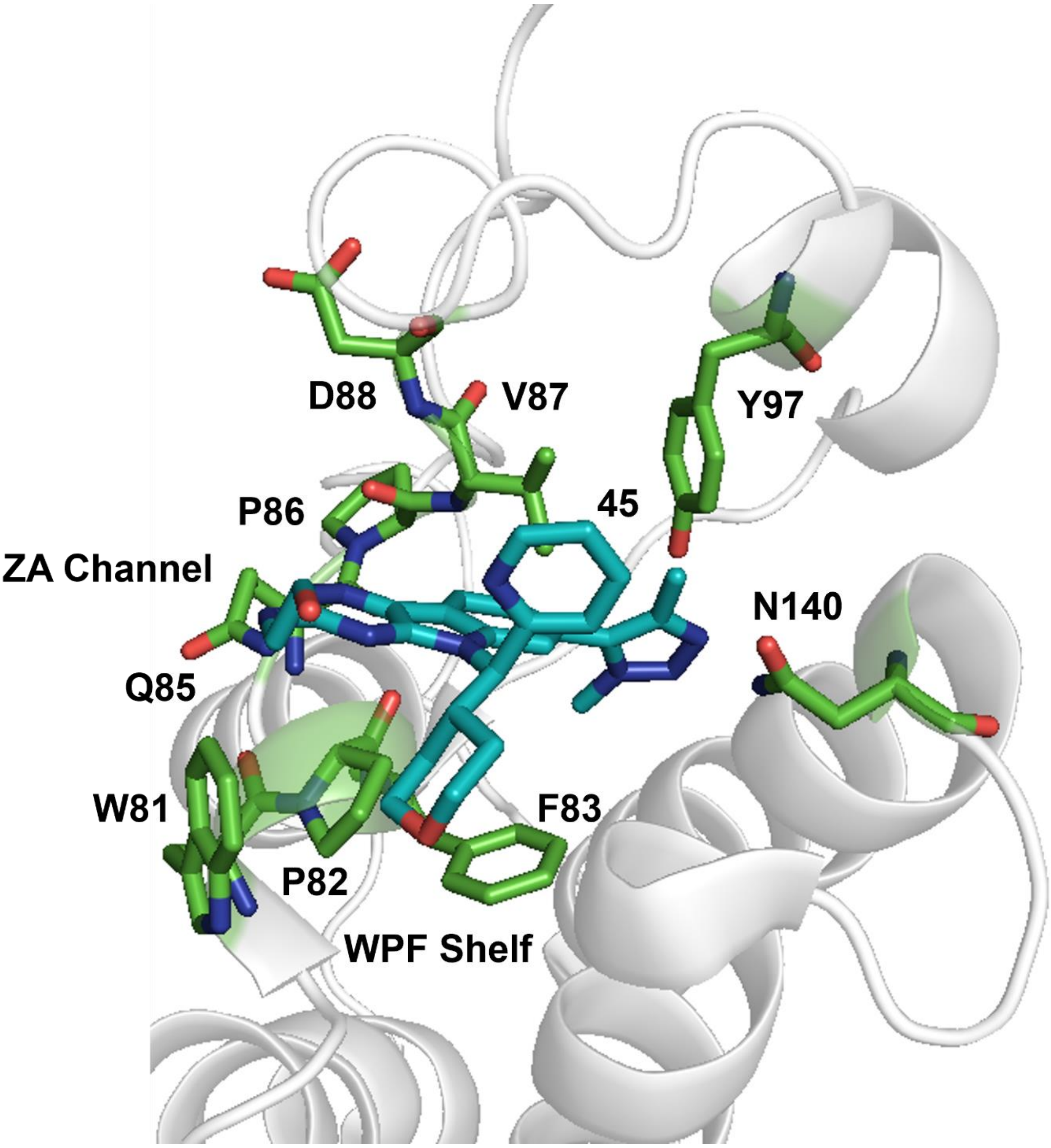

Figure S4. Binding mode of **45** in BD1 of BRD4. The receptor is shown in cartoon representation (grey), while amino acids (green) and **45** (teal) are displayed as stick models. Oxygen atoms are shown in red and nitrogen in blue. Key interaction occurs between triazole and asparagine residue in the *N*-acetylated lysine binding pocket. The triazole, pyridine and triazine moieties in **45** interact with the asparagine residue, WPF shelf and ZA channel respectively. Figure generated in PyMOL using PDB 3ZYU.[23]

References

1. Seal, J. T.; Atkinson, S. J.; Aylott, H.; Bamborough, P.; Chung, C.-w.; Copley, R. C. B.; Gordon, L.; Grandi, P.; Gray, J. R. J.; Harrison, L. A.; Hayhow, T. G.; Lindon, M.; Messenger, C.; Michon, A.-M.; Mitchell, D.; Preston, A.; Prinjha, R. K.; Rioja, I.; Taylor, S.; Wall, I. D.; Watson, R. J.; Woolven, J. M.; Demont, E. H., The Optimization of a Novel, Weak Bromo and Extra Terminal Domain (BET) Bromodomain Fragment Ligand to a Potent and Selective Second Bromodomain (BD2) Inhibitor. *J. Med. Chem.* **2020,** *63* (17), 9093-9126.
2. Inhibitors of bruton's tyrosine kinase. US20150158865A1, 2015.
3. Substituted 5-(3,5-dimethylisoxazol-4-yl)indoline-2-ones. WO2014173241A1, 2014.
4. Vine, K. L.; Locke, J. M.; Ranson, M.; Pyne, S. G.; Bremner, J. B., In vitro cytotoxicity evaluation of some substituted isatin derivatives. *Bioorg. Med. Chem.* **2007,** *15* (2), 931-938.
5. Babu, K. N.; Kinthada, L. K.; Pratim Das, P.; Bisai, A., Cu(ii)–tBu–PHOX catalyzed enantioselective malonate addition onto 3-hydroxy 2-oxindoles: application in the synthesis of dimeric pyrroloindoline alkaloids. *Chem. Commun.* **2018,** *54* (57), 7963-7966.
6. Gavai, A. V.; Norris, D.; Delucca, G.; Tortolani, D.; Tokarski, J. S.; Dodd, D.; O'Malley, D.; Zhao, Y.; Quesnelle, C.; Gill, P.; Vaccaro, W.; Huynh, T.; Ahuja, V.; Han, W.-C.; Mussari, C.; Harikrishnan, L.; Kamau, M.; Poss, M.; Sheriff, S.; Yan, C.; Marsilio, F.; Menard, K.; Wen, M.; Rampulla, R.; Wu, D.-R.; Li, J.; Zhang, H.; Li, P.; Sun, D.; Yip, H.;

Traeger, S. C.; Zhang, Y.; Mathur, A.; Zhang, H.; Huang, C.; Yang, Z.; Ranasinghe, A.; Everlof, G.; Raghavan, N.; Tye, C. K.; Wee, S.; Hunt, J. T.; Vite, G.; Westhouse, R.; Lee, F. Y., Discovery and Preclinical Pharmacology of an Oral Bromodomain and Extra-Terminal (BET) Inhibitor Using Scaffold-Hopping and Structure-Guided Drug Design. *J. Med. Chem.* **2021,** *64* (19), 14247-14265.

7. El Ashry, E. S. H.; Ramadan, E. S.; Hamid, H. M. A.; Hagar, M., Microwave Irradiation for Accelerating each Step for the Synthesis of 1,2,4-Triazino[5,6-b]indole-3-thiolsand their Derivatives from Isatin and 5-Chloroisatin. *Synlett* **2004,** *2004* (04), 723-725.

8. Patel, D. V.; Patel, N. R.; Kanhed, A. M.; Teli, D. M.; Patel, K. B.; Gandhi, P. M.; Patel, S. P.; Chaudhary, B. N.; Shah, D. B.; Prajapati, N. K.; Patel, K. V.; Yadav, M. R., Further Studies on Triazinoindoles as Potential Novel Multitarget-Directed Anti-Alzheimer's Agents. *ACS Chem. Neurosci.* **2020,** *11* (21), 3557-3574.

9. Liu, R. L. Design, synthesis, and biological evaluation of PqsR antagonists guided by classic hit-to- lead optimisation process and fragment- based methods for the treatment of Pseudomonas aeruginosa infections. PhD Thesis, University of Nottingham, 2021.

10. Allwood, D. M.; Blakemore, D. C.; Ley, S. V., Preparation of Unsymmetrical Ketones from Tosylhydrazones and Aromatic Aldehydes via Formyl C–H Bond Insertion. *Org. Lett.* **2014,** *16* (11), 3064-3067.

11. Zhou, X.; Guo, L.; Zhang, H.; Xia, R. Y.; Yang, C.; Xia, W., Nickel-Catalyzed Reductive Acylation of Carboxylic Acids with Alkyl Halides and N-

Hydroxyphthalimide Esters Enabled by Electrochemical Process. *Adv. Synth. Catal.* **2022,** *364* (9), 1526-1531.

12. Nie, F.-Y.; Cai, Y.-P.; Song, Q.-H., Visible Light-Driven Decarboxylative Alkylation of Aldehydes via Electron Donor–Acceptor Complexes of Active Esters. *J. Org. Chem.* **2022,** *87* (2), 1262-1271.

13. Morris, D. J.; Hayes, A. M.; Wills, M., The "Reverse-Tethered" Ruthenium (II) Catalyst for Asymmetric Transfer Hydrogenation: Further Applications. *J. Org. Chem.* **2006,** *71* (18), 7035-7044.

14. Substituted 2-amino-pyrazolyl-[1,2,4]triazolo[1,5a] pyridine derivatives and use thereof. WO2020215094A1, 2020.

15. Boit, T. B.; Mehta, M. M.; Kim, J.; Baker, E. L.; Garg, N. K., Reductive Arylation of Amides via a Nickel-Catalyzed Suzuki–Miyaura-Coupling and Transfer-Hydrogenation Cascade. *Angew. Chem., Int. Ed.* **2021,** *60* (5), 2472-2477.

16. Glucokinase activators. WO2007053345A1, 2007.

17. Sunwoo, K.; Won, M.; Ko, K.-P.; Choi, M.; Arambula, J. F.; Chi, S.-G.; Sessler, J. L.; Verwilst, P.; Kim, J. S., Mitochondrial Relocation of a Common Synthetic Antibiotic: A Non-genotoxic Approach to Cancer Therapy. *Chem* **2020,** *6* (6), 1408-1419.

18. Imidazo-triazine derivatives as pde10 inhibitors. WO2014177977A1, 2014.

19. Tricyclic compounds as anticancer agents. WO2022156757A1, 2022.

20. Tricyclic compound for bromodomain-containing protein inhibitor and preparation, pharmaceutical composition, and application thereof. EP3412669A1, 2017.

21. Clark, P. R.; Williams, G. D.; Hayes, J. F.; Tomkinson, N. C. O., A Scalable Metal-, Azide-, and Halogen-Free Method for the Preparation of Triazoles. *Angew. Chem., Int. Ed.* **2020,** *59* (17), 6740-6744.

22. Gianella-Borradori, M.; Christou, I.; Bataille, C. J. R.; Cross, R. L.; Wynne, G. M.; Greaves, D. R.; Russell, A. J., Ligand-based virtual screening identifies a family of selective cannabinoid receptor 2 agonists. *Bioorg. Med. Chem.* **2015,** *23* (1), 241-263.

23. Dawson, M. A.; Prinjha, R. K.; Dittmann, A.; Giotopoulos, G.; Bantscheff, M.; Chan, W.-I.; Robson, S. C.; Chung, C.-w.; Hopf, C.; Savitski, M. M.; Huthmacher, C.; Gudgin, E.; Lugo, D.; Beinke, S.; Chapman, T. D.; Roberts, E. J.; Soden, P. E.; Auger, K. R.; Mirguet, O.; Doehner, K.; Delwel, R.; Burnett, A. K.; Jeffrey, P.; Drewes, G.; Lee, K.; Huntly, B. J. P.; Kouzarides, T., Inhibition of BET recruitment to chromatin as an effective treatment for MLL-fusion leukaemia. *Nature* **2011,** *478* (7370), 529-533.
